# Evidence-Based Network Modelling to Simulate Nucleus Pulposus Multicellular Activity in Different Nutritional and Pro-Inflammatory Environments

**DOI:** 10.3389/fbioe.2021.734258

**Published:** 2021-11-10

**Authors:** L. Baumgartner, A. Sadowska, L. Tío, M. A. González Ballester, K. Wuertz-Kozak, J. Noailly

**Affiliations:** ^1^ BCN MedTech, Department of Information and Communication Technologies, Universitat Pompeu Fabra, Barcelona, Spain; ^2^ Department of Health Sciences and Technology, Institute for Biomechanics, ETH Zurich, Zurich, Switzerland; ^3^ IMIM (Hospital del Mar Medical Research Institute), Barcelona, Spain; ^4^ ICREA, Barcelona, Spain; ^5^ Department of Biomedical Engineering, Rochester Institute of Technology (RIT), Rochester, NY, United States; ^6^ Schön Clinic Munich Harlaching, Spine Center, Academic Teaching Hospital and Spine Research Institute of the Paracelsus Medical University Salzburg (Austria), Munich, Germany

**Keywords:** intervertebral disc degeneration, multicellular systems, cell activity, inflammation, *in vitro* experiments, evidence-based simulations, multifactorial environment, network modelling

## Abstract

Initiation of intervertebral disc degeneration is thought to be biologically driven. This reflects a process, where biochemical and mechanical stimuli affect cell activity (CA) that compromise the tissue strength over time. Experimental research enhanced our understanding about the effect of such stimuli on different CA, such as protein synthesis or mRNA expression. However, it is still unclear how cells respond to their native environment that consists of a “cocktail” of different stimuli that might locally vary. This work presents an interdisciplinary approach of experimental and *in silico* research to approximate Nucleus Pulposus CA within multifactorial biochemical environments. Thereby, the biochemical key stimuli glucose, pH, and the proinflammatory cytokines TNF-α and IL1β were considered that were experimentally shown to critically affect CA. To this end, a Nucleus Pulposus multicellular system was modelled. It integrated experimental findings from *in vitro* studies of human or bovine Nucleus Pulposus cells, to relate the individual effects of targeted stimuli to alterations in CA. Unknown stimulus-CA relationships were obtained through own experimental 3D cultures of bovine Nucleus Pulposus cells in alginate beads. Translation of experimental findings into suitable parameters for network modelling approaches was achieved thanks to a new numerical approach to estimate the individual sensitivity of a CA to each stimulus type. Hence, the effect of each stimulus type on a specific CA was assessed and integrated to approximate a multifactorial stimulus environment. Tackled CA were the mRNA expressions of Aggrecan, Collagen types I & II, MMP3, and ADAMTS4. CA was assessed for four different proinflammatory cell states; non-inflamed and inflamed for IL1β, TNF-α or both IL1β&TNF-α. Inflamed cell clusters were eventually predicted in a multicellular 3D agent-based model. Experimental results showed that glucose had no significant impact on proinflammatory cytokine or ADAMTS4 mRNA expression, whereas TNF-α caused a significant catabolic shift in most explored CA. *In silico* results showed that the presented methodology to estimate the sensitivity of a CA to a stimulus type importantly improved qualitative model predictions. However, more stimuli and/or further experimental knowledge need to be integrated, especially regarding predictions about the possible progression of inflammatory environments under adverse nutritional conditions. Tackling the multicellular level is a new and promising approach to estimate manifold responses of intervertebral disc cells. Such a top-down high-level network modelling approach allows to obtain information about relevant stimulus environments for a specific CA and could be shown to be suitable to tackle complex biological systems, including different proinflammatory cell states. The development of this methodology required a close interaction with experimental research. Thereby, specific experimental needs were derived from systematic *in silico* approaches and obtained results were directly used to enhance model predictions, which reflects a novelty in this research field. Eventually, the presented methodology provides modelling solutions suitable for multiscale approaches to contribute to a better understanding about dynamics over multiple spatial scales. Future work should focus on an amplification of the stimulus environment by integrating more key relevant stimuli, such as mechanical loading parameters, in order to better approximate native physiological environments.

## Introduction

Intervertebral disc degeneration is a major cause of low back pain, a disability that stands for one of the highest health burdens worldwide (Hoy et al., 2014). The intervertebral disc is avascular and consists of three specialized tissues: the Nucleus Pulposus (NP), a proteoglycan-rich and highly hydrated structure in the center of the disc, the Annulus Fibrosus, a juxtaposition of concentric fibrous lamellae that surrounds the NP, and the Cartilage Endplate, a thin layer of hyaline cartilage that separates the NP and the inner Annulus Fibrosus from the vertebral bodies. In each tissue, specialized cells regulate the synthesis of a finely balanced extracellular matrix (ECM) by synthesizing tissue proteins and proteases according to a “cocktail” of mechanical and biochemical stimuli sensed by the cells (reviewed in Baumgartner et al., 2021). Thanks to its specialized structure and composition, the intervertebral disc has a very high strength and classical tissue injury might happen at internal pressures higher than 10 MPa (Veres et al., 2008). Thus, organ failure is most likely a slow process, triggered by an adverse cell (micro-) environment, leading to altered cell activity (CA) that finally compromises the tissue composition and strength. These mechanisms, where compromised CA occur in response to undue biochemical and/or mechanical cues, among others, are cornerstone in injury processes. We hereby refer to these mechanisms as biologically-driven injury mechanisms.

Over the past decades, experimental studies have investigated the impact of a broad variety of stimuli on NP CA. In addition to mechanoregulatory stimuli, biochemical stimuli influence NP CA, whereby nutrition-related stimuli and proinflammatory cytokines have been investigated in most depth. The importance of nutrition-related stimuli is a consequence of the avascularity of the disc, where nutrient supply to the cells is diffusion-dependent. Consequently, gradients of pH and glucose (glc) concentration emerge between the peripheral vascular beds at the vertebral endplates and the mid-transversal plane of the NP (Urban et al., 2004). The likely consequences of these gradients in the mechanically loaded intervertebral disc were captured by quantitative *in silico* explorations (Malandrino et al., 2015; Baumgartner et al., 2021). However, approximations of individual cell responses at the (multi-) cellular level remain poorly investigated. At the micro-/nanoscale level, cell environments are heterogenous, i.e. local cellular stimulus environments vary, e.g. due to local, proinflammatory cytokine expression. Proinflammatory cell stimulations were pointed out as possible key factors in the catabolic shift of NP CA, and might contribute to the development of different degenerative phenotypes, e.g., herniated vs. non-herniated discs (Risbud and Shapiro, 2014; Le Maitre et al., 2007; Johnson et al., 2015). Special focus was thereby set on the proinflammatory cytokines interleukin 1 beta (IL1β) and tumor necrosis factor alpha (TNF-α), which have the potential to alter CA by activating intracellular signaling pathways such as Notch, JNK or NF-κB (Baumgartner et al., 2021). In agreement with that, it could be shown that the amount of cells immunopositive for IL1β and TNF-α rises as intervertebral disc degeneration progresses (Le Maitre et al., 2005; Le Maitre et al., 2007).

In order to cope with the overwhelming complexity of the intracellular pathways and interactions thereof while enabling interpretable representations of multifactorial cell regulation, high-level physiological modelling is particularly appealing. A new modelling approach was recently proposed in intervertebral disc systems biology, focusing on the multicellular level where stimuli identified to be relevant for NP cell regulations were directly linked to CA (Baumgartner et al., 2020). Whereas the cell per-se was considered as a black box, this methodology admitted biological data as inputs, to approximate the integration of the effects of individual stimuli on the effective CA in multifactorial environments. Hence, *in vitro* studies were used to provide detailed information about the relationship between different stimulus concentrations and a corresponding CA. Results were subsequently integrated to estimate effective CA in multifactorial biochemical environments that would be closer to the reality of native tissues. However, to approach cell responses within native tissues the pre-processing of biological evidence for proper and systematic integration into systems biology models requires further investigation.

It could be experimentally shown that CA is influenced 1) by the concentration of a stimulus within the cellular (micro-) environment, and 2) by the type of a stimulus, i.e., the effect of different stimulus concentrations affect different mRNA expression in a different way, e.g., Rinkler et al., 2010; Neidlinger-Wilke et al., 2012; Gilbert et al., 2016. In our recent work, we addressed the interpretable modelling and simulation of the combined effects of different stimulus concentrations on NP CA (Baumgartner et al., 2020). The stimuli we included were glc, pH and IL1β, and the CA studied were the mRNA expressions of Aggrecan (Agg), Collagen Types I & II (Col-I, Col-II) (the main ECM components), and MMP3 and ADAMTS4 (key proteases involved in tissue degradation). The simulated multicellular environment was represented through an agent-based (AB) model and consisted of non-inflamed and IL1β-inflamed NP cells. Normalized mRNA expressions were estimated, depending on the predicted cell states (CS) in terms of immunopositivity (non-inflamed; inflamed).

Considering that the impact of a stimulus on a CA does not only depend on the stimulus concentration, but also on the sensitivity of the CA to that stimulus type (e.g., IL1β proinflammatory cytokines might not have the same effect on MMP3 mRNA expression as TNF-α proinflammatory cytokines), we hypothesize that further modelling parameters are necessary to reflect this sensitivity and improve numerical predictions, through a better integration of experimental data. Hence, this publication is a methodological article that reports on a new enabling technology to approximate the integrative effects of multifactorial environments on disc cell stimulation within the NP. Moreover, experimental research was conducted, specifically designed based on modelling requirements, to gain additional evidences about the effect of glc and TNF-α on CA. Based on these new evidences, the modelling of the proinflammatory environment was extended.

## Methods

### Methodological Approach – Overview

**FIGURE 1:**
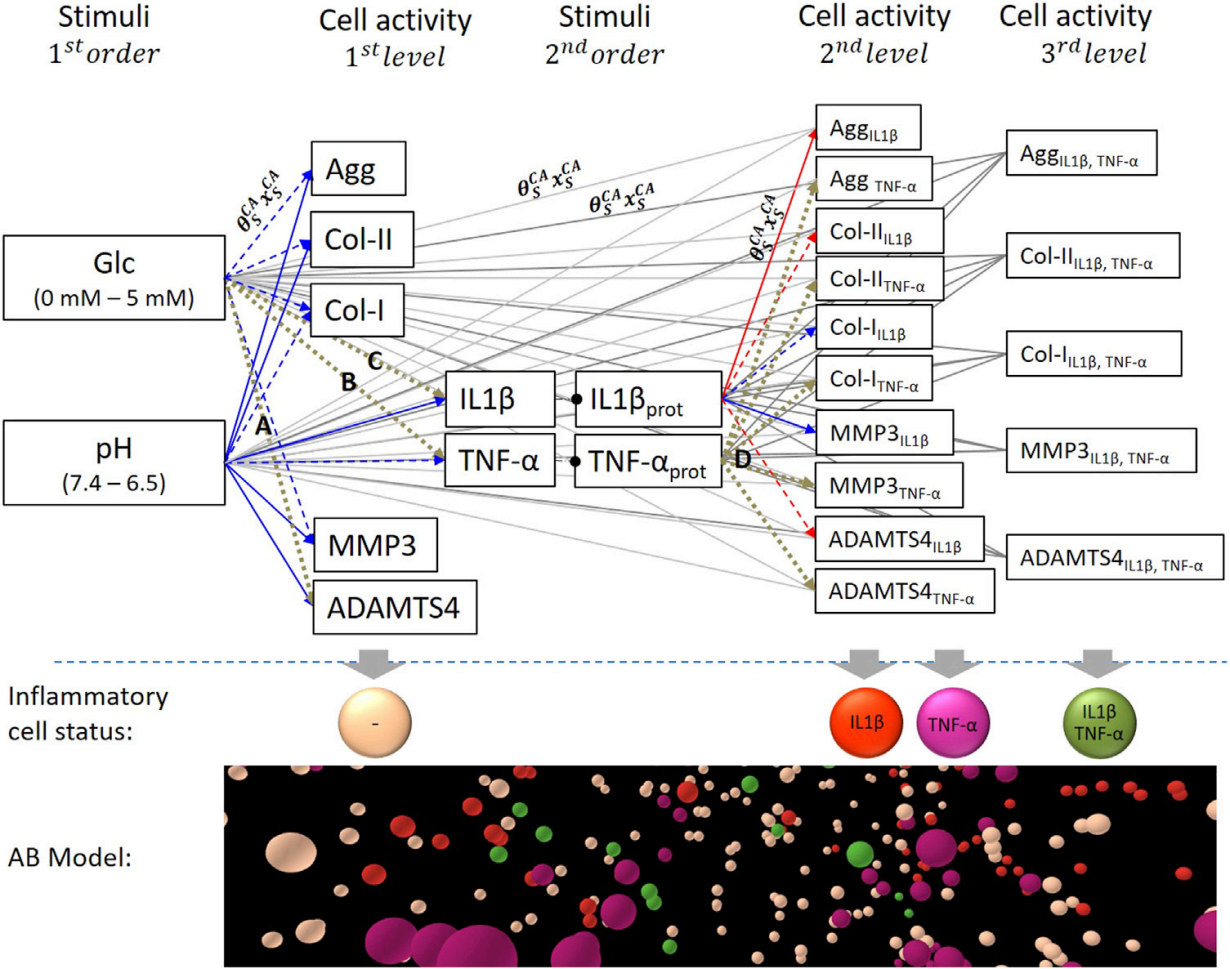
System of interest based on nutrition-related stimuli glucose (glc) and pH. Stimulus – cell activity (S-CA) relationships were either activating **(blue)** or inhibiting **(red)** according to experimental findings. Dashed blue and red arrows marked non-significant (*p* > 0.05) relationships according to experimental findings and brown dotted arrows unknown S-CA relationships. Respective connections between 1st order stimuli and 2nd/3rd level CA and 2nd order stimuli and 3rd level CA were represented as grey lines to provide a better visibility. Each S-CA relationship is determined by the sensitivity of a CA to a stimulus type (weighting factor, $\theta_{S}^{CA})$ and by the sensitivity of a CA to a stimulus concentration ( $x_{S}^{CA})$ (exemplarily illustrated within the system of interest). Resulting CA for different inflammatory cell states were calculated and displayed within a 3D Agent-based (AB) model.

The computational model of the system of interest included the nutrition-related stimuli glc, pH and the proinflammatory cytokines TNF-α and IL1β, as regulatory variables able to lead to four different proinflammatory CS; 1) non-inflamed cells, cells immunopositive for 2) IL1β or 3) TNF-α or 4) for both IL1β&TNF-α. For each CS, targeted CA were the mRNA expressions of the key tissue proteins Agg, Col-I, Col-II and proteases MMP3, and ADAMTS4 (Figure 1).

Glc concentration and pH are user-defined (1st order stimuli) and regulate TNF-α and IL1β proinflammatory cytokine expressions (2nd order stimuli). Glc concentration and pH values could vary in physiologically relevant ranges of 0–5 mM glc and a pH 6.5–7.4, respectively (Rinkler et al., 2010; Gilbert et al., 2016). CA of non-inflamed cells (1st level CA) were calculated based on the nutrition-related environment, whilst CA of cells immunopositive for TNF-α and IL1β (2nd level CA) were additionally influenced by their corresponding 2nd order stimulus. Accordingly, 3rd level CA reflected cells with immunopositivity for both proinflammatory cytokines. To sum up, 1st level CA was defined by the combination of two stimuli, 2nd level CA by the combination of three stimuli and 3rd level CA by the combination of four stimuli.

Each connection between a stimulus and a CA described the individual stimulus-cell activity relationships (S-CA relationships). It was determined by the sensitivity of a CA to a stimulus type (subscript S), reflected by a weighting factor $\left(\theta_{S}^{CA}\right)$ and by the sensitivity of a CA to a certain stimulus concentration $\left(x_{S}^{CA}\right)$ (Figure 1). S-CA relationships were categorized according to their activating or inhibiting nature (blue/red arrows, Figure 1), and to their respective biological significance (continuous vs. dashed arrows, Figure 1), based on experimental evidence (Le Maitre et al., 2005; Rinkler et al., 2010; Neidlinger-Wilke et al., 2012; Gilbert et al., 2016). In Figure 1, repeated connections with the same characteristics over different CA levels, were represented as grey lines to make the network representation visually lighter. S-CA relationships that were not found in the literature (relationships A-D, Figure 1) were experimentally obtained hereby through *in-vitro* experimental data (*In-Vitro Experiments* section).

**FIGURE 2:**
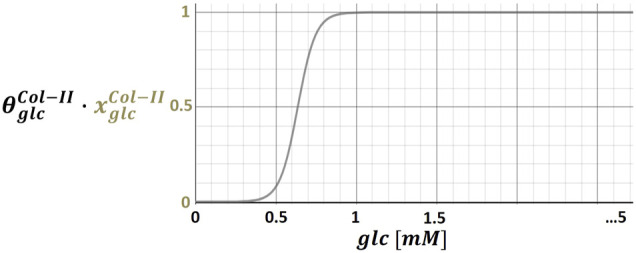
Illustration of stimulus-cell activity relationships by means of the glucose (glc) – collagen type II (Col-II) relationship; continuous functions assign physiologically relevant glc concentrations (i.e. 0 - 5 mM) to a normalized Col-II mRNA expression, which was multiplied by an individual weighting factor ( $\theta_{S}^{CA}$ ).

The data-based determination of $x_{S}^{CA}$ was previously detailed in Baumgartner et al., 2020. In short: to determine $x_{S}^{CA}$ of nutrition-related stimuli, continuous, sigmoidal functions were built based on discrete experimental findings of x-fold changes in mRNA expressions. Thereby, each stimulus concentration within a physiologically relevant range was assigned to a normalized value ( $x_{S}^{CA}$ ) that ranged from a minimum of 0 to a maximum of 1 (Figure 2).

$x_{S}^{CA}$ of proinflammatory cytokines was mathematically approximated by an inflammation submodel (see *Determination of Inflammation* section), because of a lack of information about physiological ranges of proinflammatory cytokine concentrations. Once determined, each $x_{S}^{CA}$ was multiplied by a S-CA specific weighting factor (Figure 2), the calculation of which is presented in *Determination of Weighting Factors* section.

To eventually combine the respective effects of different S-CA relationships and estimate effective CA in terms of individual mRNA expressions, a methodology was developed to semi-quantitatively predict mRNA expressions within a system of parallel networks (PN). The corresponding theoretical framework is briefly described in *Overview of the Parallel Networks Methodology* section to ensure the comprehensibility of the predicted CA.

Eventually, the CA for each CS was computed with an AB software (NetLogo, v. 6.0.2, Wilensky, 1999) (Figure 1) that integrated the network calculations with the spatial dimension of a multicellular system. The 3D AB model mimicked a proinflammatory environment within a 1 mm³ volume of the NP environment as previously explained (Baumgartner et al., 2020). Thereby, 4,000 agents of a diameter of 10 µm were randomly distributed, representing an average cell density of NP cells (Maroudas et al., 1975). The inflammatory environment is detailed in *Determination of Inflammation* section.

### *In Vitro* Experiments

To determine the unknown S-CA relationships, the effect of glc on TNF-α, IL1β and ADAMTS4 mRNA expression (Figure 1, relationships A - C), and the effect of TNF-α on the targeted mRNA expressions (Figure 1, relationships D) were assessed through *in vitro* experiments on bovine caudal NP cells.

Experimental protocols were established by considering both, previous experimental research and *in silico* findings. To determine CA under different glc concentrations, the experimental setup was inspired by Rinkler et al., 2010, whose data were previously used to determine the glc-CA relationships within the system (Figure 1, Baumgartner et al., 2020). Accordingly, bovine NP cells were seeded into alginate beads and exposed to glc levels of either 0, 0.5 and 5 mM, whereby the 5 mM concentration served as control. Additional glc concentrations of 0.8 and 1 mM were considered to reflect hypothetical transitional nutritional conditions within the NP that might differentiate normal and early degenerated intervertebral discs, according to previous *in silico* findings (Ruiz Wills et al., 2018).

To assess the effect of TNF-α on the targeted mRNA expressions, a 5 mM glc medium was enriched with a TNF-α protein concentration of 10 ng/ml, in agreement with previous experimental research on proinflammatory cytokines (Le Maitre et al., 2005; Millward-Sadler et al., 2009; Walter et al., 2015; Likhitpanichkul et al., 2016; Yang et al., 2017). Cell cultures exposed to 5 mM glc concentration without TNF-α served as control.

In addition to the required S-CA relationships, cell viability was measured for all the conditions. The effect of glc (partial) deprivation on Agg, Col-I, Col-II and MMP3 and the effect of TNF-α on the mRNA expressions of TNF-α and IL1β was also assessed. Corresponding results are presented as Supplementary Materials S1, S2.

#### Cell Isolation and Culture

NP cells were isolated from bovine tails (*n* = 5) by 0.3% Dispase II (04942078001, Roche, Basel, Switzerland)/0.2% Collagenase NB4 (17454, Serva, Heidelberg, Germany) digestions with 3% Antibiotics-Antimycotics solution in PBS as previously described (Cambria et al., 2020; Sadowska et al., 2020). Cells were then expanded in 2D conditions for around 14 days in DMEM/F-12 (Thermo Fisher/Gibco 11320033) [25 mM glc], with 10% fetal calf serum (FCS) (F7524, Sigma) and 1% Antibiotics-Antimycotics solution. Three to 5 days prior to the experiment, the medium was changed to DMEM (Thermo Fisher/Gibco 11965092) with 10% FCS and 1% Antibiotics-Antimycotics.

#### Cell Stimulation

All cell stimulation experiments were conducted on passage 2 NP cells seeded into alginate beads as previously described (Krupkova et al., 2014). Briefly, NP cells were transferred to a 1.2% alginic acid sodium salt (180947, Sigma-Aldrich, St. Louis, MO, United States) at a density of 4 × $10^{6}$ cells per ml alginate (reflecting the average cell density within the NP Maroudas et al., 1975). A 21 G needle was used to create the alginate beads. Eventually, an average of 101 ± 8 alginate beads with a total of 8.5–9 × $10^{6}$ NP cells was obtained from each donor. Beads were cultured in 5 mM glc for 24 h, to allow the cells to adapt their glycogen stores to a physiological glc environment. Subsequently, each well of a six well plate was exposed for 48 h to one of the aforementioned glucose concentrations or to a TNF-α enriched medium (10 ng/ml human recombinant TNF-α (17.4 kDa, PeproTech, 300-01A)), at 5 mM glc, under a normoxic environment and pH 7.4 . The different glc concentrations were created by mixing DMEM high glc [Thermo Fisher (Gibco) 11965092] and DMEM no glc [Thermo Fisher (Gibco) 11966025] in the respective ratios. The culture medium was changed after 24 h in order to maintain the chosen glc conditions under metabolic cell activity. Imposed culture conditions were static and mRNA expression and cell viability were assessed immediately after the 48 h of exposure to the stimulus.

#### Cell Viability Measurement and mRNA Expression Analysis

Cell viability was assessed by exposing one bead per condition to a 10 μm Calcein AM (CaAM)/1 μM Ethidium Homodimer (EthHD) solution, for approximately 1 h. Afterwards, the bead was gently squeezed between a microscope slide and its cover glass, and cells were counted under a fluorescence microscope (Olympus IX51, Tokyo, Japan). The number of cells was analyzed within up to four different regions of the bead, and cells were counted within a predefined area, using a grid of constant size for each sample. Remaining alginate beads were dissolved during 30 min and occasional shaking in a dissolving buffer (55 mM Sodium citrate solution (71406, Sigma, in 0.9% NaCl)). Isolated cells were pelleted by centrifugation, washed 1× with PBS and subsequently lysed in the specific lysis RLT buffer (plus 1% 2-Mercaptoethanol) of the RNeasy Mini Kit 50 (QIAGEN, ID 74104). mRNA was extracted following the protocol provided by the manufacturer, and the quality and quantity of RNA was analyzed using a Nanodrop 1,000 Spectrophotometer (Thermo Fisher Scientific). 1 µg of total RNA was finally reverse transcribed into cDNA in a 30 µl volume using the Taqman Reverse Transcription kit (#4374966, Applied Biosystems, United States).

cDNA was then mixed with Bovine TaqMan primers (Primer Seq. No. ADAMTS4: Bt03224693_m1, MMP3: Bt04259497_m1, Agg: Bt03212186_m1, Col-I: Bt03214883_m1, Col-II, Bt03251861_m1) to assess changes in the gene expressions of Agg, Col-I, Col-II, ADAMTS4 and MMP3. As for TNF-α and IL1β gene expressions, cDNA was additionally amplified, as initial real-time qPCR showed a gene expression at high Cq. Amplification was performed following the manufacturer’s protocol. In short, cDNA was mixed with TaqMan PreAmp Master Mix (2X) (#4391128, Thermo Fisher, Switzerland) and pooled assay mix consisting of TaqMan Primers (Thermo Fisher, Switzerland) diluted with 1X TE Buffer (AM9849, Thermo Fisher, Switzerland) to a final concentration of 0.2X. For the gene expression analysis 4.5 µL or 37.5 ng of amplified cDNA was combined with 5 µL TaqMan Fast Universal PCR Master Mix (2X) (#4352042, Thermo Fisher, Switzerland) and 0.5 µL TaqMan primers (Life Technology, Primer Seq. No: TNF-α: Bt03259156_m1, IL1β: Bt03212741_m1) to a total volume of 10 µL per well.

Gene expressions were measured by the real-time qPCR (CFX96 Touch™ Detection System, Biorad) and all conducted in duplicate. Previous testing revealed YWHAZ (TaqMan Primer Seq. No: Bt01122444_g1) as an appropriate housekeeping gene. The $-2^{\Delta \Delta Ct}$ method was used to normalize and compare the mRNA contents between treatments and the control sample (5 mM glc).

#### Statistics

Statistical analyses were performed using SPSS software version 23.0. Evaluations were done on the ΔCt values, i.e., on the difference of the targeted genes to the housekeeping gene, leading to statistically reliable data by obtaining a variance as well for control groups. Based on the small sample sizes, non-parametric tests were performed, consisting of a Kruskall-Wallis H test for the evaluation of the effect of different glc concentrations on mRNA expressions, and a Mann-Whitney *U* test to evaluate the effect of a TNF-α enriched medium. The significance level was set to *p* < 0.05.

### Overview of the Parallel Networks Methodology

To mathematically provide interrelated results for many parallel networks, a methodology was developed to 1) estimate the activation of each CA by integrating the effect of each corresponding S-CA relationship and 2) to relate the activation of each CA to other concurrent CA. A network was defined as the group of S-CA relationships that converges to a specific CA. From now on, the methodology hereby defined is referred to as the parallel networks (PN)-Methodology. It required the predefinition of a system, i.e., the PN-system, of all the CA where a relative interpretation is desired. In the system of interest presented in Figure 1, these CA would be the 1st, 2nd and 3rd level CA.

To calculate a PN-system, an equation was developed, referred to as the PN-equation Eq. 1.

$$\omega_{CA,CS}=\left(\left(\frac{1+\sum \theta_{\alpha }}{\sum \theta_{\alpha }}\right)\left(\frac{\sum \theta_{S,\alpha }^{CA}x_{S,\alpha }^{CA}}{1+\sum \theta_{S,\alpha }^{CA}x_{S,\alpha }^{CA}}\right)\right)\cdot \left(1-\left(\left(\frac{\sum \theta_{S,\beta }^{CA}}{\sum \theta_{S,\alpha }^{CA}\;+\;\sum \theta_{S,\beta }^{CA}}\right)\left(\left(\frac{1+\sum \theta_{\beta }^{CA}}{\sum \theta_{\beta }^{CA}}\right)\left(\frac{\sum \theta_{S,\beta }^{CA}x_{S,\beta }^{CA}}{1+\sum \theta_{S,\beta }^{CA}x_{S,\beta }^{CA}}\right)\right)\right)\right)$$ (1)

The PN-equation originated from the graph-based modelling approach developed by Mendoza and Xenarios, 2006 that semi-qualitatively describes biological network dynamics at a subcellular scale, with integration of the simultaneous effects of different inputs on the effective regulation of a specific node. Accordingly, the overall activation of a CA of a certain CS, $\omega_{CA,CS}$ , in Eq. 1, was determined by an activating (subscripts α ) and an inhibiting (subscripts β) term. Thereby, $\theta_{\alpha }$ are the weighting factors of all activating S-CA relationships within the PN-system, and $\theta_{S,\alpha }^{CA}\;$ and $\theta_{S,\beta }^{CA}$ are respectively the activating and inhibiting weighting factors of a specific network. Finally, $\theta_{\beta }^{CA}$ reflects all the inhibiting connections within the same CA, independently of the CS. $\omega_{CA,CS}$ were bound between 0–1 and reflect PN-activities. These provide activation levels for the individual, interrelated CA within the PN-system. Hence, the PN-activity is a quantity that assesses the CA. Accordingly, the lower a PN-activity is, the lower the activity of a cell to express that respective mRNA.

The PN-activity is a scalar calculated with 4 decimals, determined based on pilot network calculations. The resolution was aimed to be sensitive enough to reflect small changes in CA, which were often identified within three to four decimal places (see Figure 10 in the *Results* section). Such resolution makes sense with regard to the long-term cumulative effect of small persistent perturbations, as it is likely to happen in slowly developing disorders such as intervertebral disc degeneration. Accordingly, the continuous functions formerly determined (Baumgartner et al., 2020) to define the sensitivity of a CA to a stimulus concentration (briefly explained in the *Methodological Approach–Overview* section) were refined to achieve this resolution. Functions are provided as Supplementary Material S3.

### Determination of Weighting Factors

**FIGURE 3:**
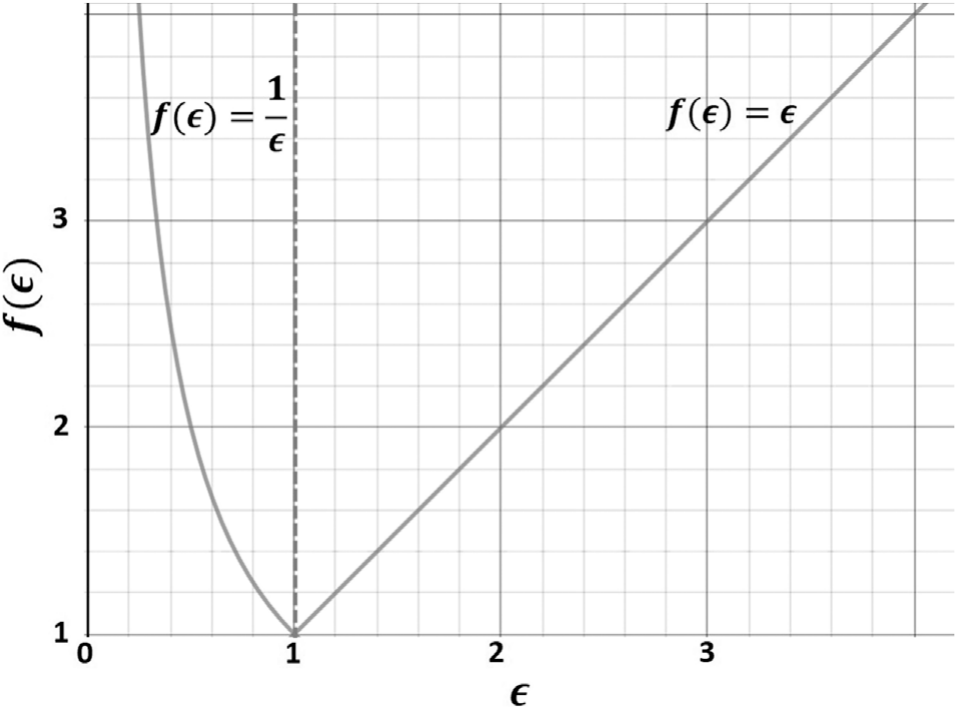
Cellular effort (*f*(*ϵ*)) to compare augmentations and decreases of x-fold mRNA expressions ( ϵ ).

To determine individual weighting factors ,  experimental information about the capacity of a stimulus to alter CA was used. This capacity is reflected by the maximal change in x-fold mRNA expression ( ϵ ) found within the physiologically relevant range of stimulus concentrations. Any change induced by a varying stimulus concentration led to x-fold mRNA expressions either higher ( ϵ>1 ) or lower ( 0<ϵ<1 ) than the control level (1). To mathematically achieve semi-bounded ranges for both increase and decrease of x-fold mRNA, reciprocal proportional relationships, f(ϵ)=ϵ and $f\left(\epsilon \right)=\frac{1}{\epsilon }$ , were implemented for ϵ>1 and for 0<ϵ<1 , respectively (Figure 3). As such, f(ϵ) , from now on called the “cellular effort,” becomes infinite for both increased and decreased mRNA expressions relative to control. Note that the wording “cellular effort” does not refer to any biological intracellular activity here.

**FIGURE 4:**
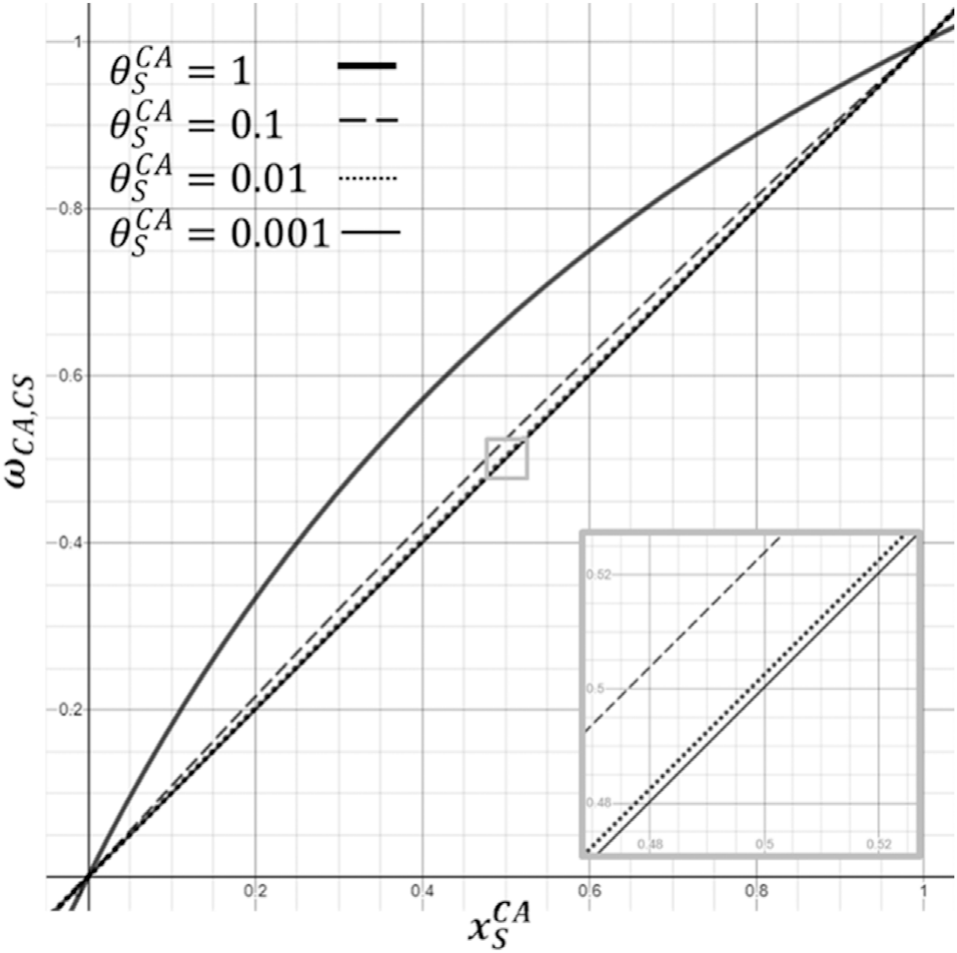
Illustration of effect of the size of a weighting factor ( $\theta_{S}^{CA}$ ) of the sensitivity of a CA to a certain stimulus concentration ( $x_{S}^{CA}$ ) on the overall cell activity $\left(\omega_{CA,CS}\right)$ . Example of different values ranging from $\theta_{S}^{CA}=0.001$ to $\theta_{S}^{CA}=1$ .

To obtain $\theta_{S}^{CA}$ , f(ϵ) was scaled by a constant scaling factor ( $\vartheta_{\theta_{max}}$ ) (Eq. 2) to a predefined range of $0.01\leq \theta_{S}^{CA}\leq 1$ . Hence $\theta_{min}$ = 0.01, $\theta_{max}=1$ and, accordingly $\vartheta_{\theta_{max}}=\;\vartheta_{1}$ . Values of 0.01 (or lower) approximate a linear coupling between $x_{S}^{CA}$ and $\omega_{CA,CS}$ (Mendoza and Xenarios, 2006) (Figure 4).

$$f\left(\epsilon \right)/\vartheta_{\theta_{max}}=\;\theta_{S}^{CA}$$ (2)

If a stimulus type did not significantly alter an x-fold mRNA expression, $\theta_{S}^{CA}\;$ was set to 0.01, approximating a linear relationship between $x_{S}^{CA}$ and $\omega_{CA,CS}$ . Experimental data about x-fold mRNA expressions was obtained from literature and from the actual study (see *Results and Experimental Results and System of Interest* sections) (Table 1).

**TABLE 1:** Individual weighting factors for the tackled PN-system, i.e., 1st, 2nd and 3rd level CA (Figure 1), along with the scaling factors and cellular efforts. Individual weighting factors were derived from the cellular effort [ f(ϵ) ], based on x-fold mRNA expressions ( ϵ ). The scaling factor $\vartheta_{\theta_{max}}$ = $\vartheta_{1}=28.7$ was determined by the S-CA relationship pH-MMP3.

Stimulus	mRNA	ϵ	f(ϵ)	$\theta_{S}^{\mathrm{CA}}\left(\;\vartheta_{1}=28.7\right)$	Source	Cell type
Glc	Agg	NS, act	—	0.0100	Rinkler et al. (2010)	human
	Col-I	NS, act	—	0.0100	Rinkler et al. (2010)	human
	Col-II	NS, act	—	0.0100	Rinkler et al. (2010)	human
	MMP3	NS, act	—	0.0100	Rinkler et al. (2010)	human
	ADAMTS4	NS, act	—	0.0100	Actual study	bovine
pH	Agg	0.37	2.7027	0.0942	Gilbert et al. (2016)	human
	Col-I	NS, act	—	0.0100	Gilbert et al. (2016)	human
	Col-II	0.63	1.5873	0.0553	Neidlinger-Wilke et al. (2012)	bovine
	MMP3	28.7	28.7000	1.0000	Gilbert et al. (2016)	human
	ADAMTS4	5.7	5.7000	0.1986	Gilbert et al. (2016)	human
IL1β	Agg	0.45^a^	2.2222	0.0774	Le Maitre et al. (2005)	human
	Col-I	NS, act	—	0.0100	Le Maitre et al. (2005)	human
	Col-II	NS, inh	—	0.0100	Le Maitre et al. (2005)	human
	MMP3	10.8^a^	10.8000	0.3763	Le Maitre et al. (2005)	human
	ADAMTS4	NS, inh	—	0.0100	Le Maitre et al. (2005)	human
TNF-α	Agg	NS, inh	—	0.0100	Actual study	bovine
	Col-I	0.31	3.2258	0.1124	Actual study	bovine
	Col-II	0.06	16.6667	0.5807	Actual study	bovine
	MMP3	26.85	26.8500	0.9355	Actual study	bovine
	ADAMTS4	5.77	5.7700	0.2010	Actual study	bovine

NS: Not significant; act: activating; inh: inhibiting.

a Estimated ϵ .

To explore the impact of individualized weighting factors on a PN-activity, the respective effects of three physiologically relevant nutritional environments in terms of pH and glc concentrations were calculated: one optimal nutritional environment (Nachemson, 1969; Rinkler et al., 2010), and two altered nutritional environments in the mid-transverse plane. These two mid-transverse plane environments were defined through our in-house mechanotransport finite element (FE) simulations (Ruiz Wills et al., 2018) and referred to the anterior region of the NP where the most adverse nutrient conditions arose within the mechanically loaded intervertebral disc. They respectively reflected glc concentration and pH values for 1) non-degenerated and 2) early degenerated cartilage endplate conditions. The nutrient concentrations around the mid-transverse plane of a non-degenerated mechanically loaded intervertebral disc were referred to as borderline conditions (Table 2).

**TABLE 2:** Nutrition-related stimuli, input parameters.

	Optimal conditions	Borderline conditions	Early degenerated conditions
Glucose [mM]	5	1.0293	0.8901
pH	7.1	6.9531	6.9349

A second set of calculations was run with all the weighting factors set to 0.01, in order to assess the impact of a systematic integration of stimulation strengths in the PN-system.

### Determination of Inflammation

**FIGURE 5:**
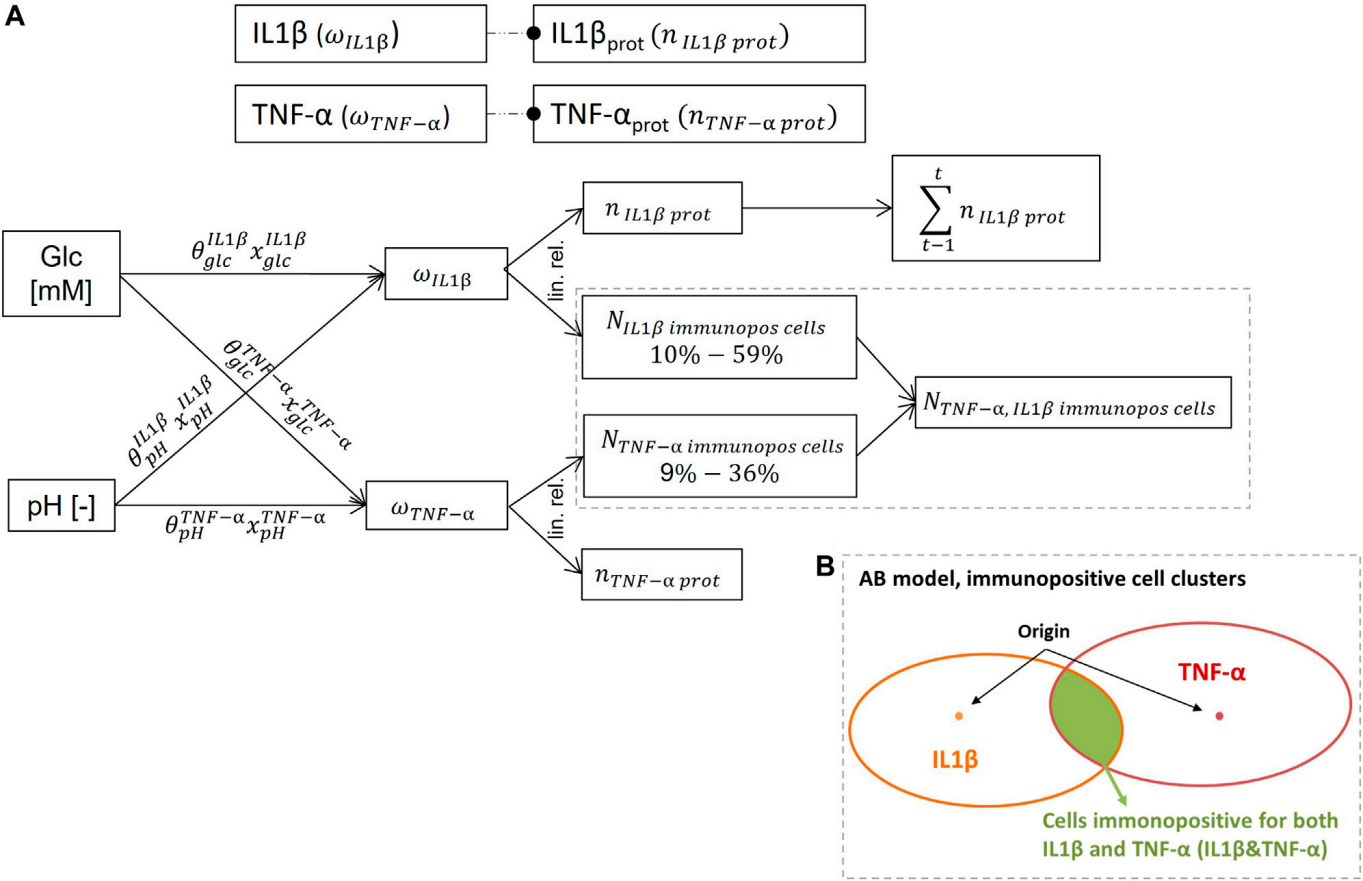
Inflammation submodel. **(A)**: underlying network of the schematically represented inflammation submodel in Figure 1 **(top)**, to approach mRNA expressions and protein synthesis of TNF-α and IL1β. **(B)**: illustration of the determination of NP cells immunpositive for both, TNF-α and IL1β by the agent-based (AB) model. Prot: protein, glc: glucose, immunopos: immunopositive, n: normalized, N: number, t: time, lin. rel.: linear relationship.

To estimate inflammatory parameters, an inflammation submodel was developed, based on previous work reported in Baumgartner et al., 2020. Based on the user-defined nutritional environment, a global (i.e., not cell-specific) normalized CA for TNF-α ( $\omega_{TNF-\alpha }$ ) and IL1β ( $\omega_{IL1\beta })$ mRNA expressions was predicted. This global normalized CA was used, moreover, to estimate the amount of immunopositive cells and concentrations of proinflammatory cytokines (Figure 5).

mRNA expressions of TNF-α and IL1β were estimated by using the regulatory network ( $\omega_{i}$ ) introduced by Mendoza and Xenarios, 2006, and they were allowed to vary within a normalized range, i.e., from 0 to 1. The proinflammatory cytokine synthesis was programmed to be proportional to the corresponding mRNA expression. The half-life of IL1β proteins was set to 2 h (Baumgartner et al., 2020), whereas a half-life of 1 h was imposed for TNF-α chosen according to the distantly related data of Oliver et al., 1993. 1 h corresponded to the time-step of our AB model (Baumgartner et al., 2020) and was, therefore, aligned with the shortest implementable half-life.

To estimate current amounts of inflamed cells, $\omega_{IL1\beta }$ and $\omega_{TNF-\alpha }\;$ were proportionally related to the percentage of inflamed human NP cells as experimentally assessed for degenerated and non-degenerated human intervertebral discs (Le Maitre et al., 2007). Those authors found that the percentage of inflamed cells ranges within approx. 10–59% for IL1β and approx. 9–36% for TNF-α (mean values ± two standard errors). For example: the percentages of IL1β inflamed cells are in a range of 17%±7% for non-degenerated NP and in a range of 52%±7% for degenerated NP. Hence, the overall range considered for IL1β inflamed cells was 10–59%.

To initialize the immunopositivity within the AB model, 30 out of 4,000 cells were randomly selected as nucleation points for 15 IL1β and 15 TNF-α immunopositive clusters. Clusters were formed around those points according to the calculated percentage of inflamed cells consistent with current nutrient concentrations and considering the globally shortest distance from an inflamed to a non-inflamed cell. Based on the randomly chosen static position of each cell, unique forms of proinflammatory cell clusters emerged for each model setup.

The number of cells immunopositive for both IL1β&TNF-α was determined by the AB model, being the cells located in overlapping areas of IL1β and TNF-α immunopositive cell clusters (Figure 5B). Eventually, proinflammatory environments were calculated for optimal, borderline and early degenerated nutritional conditions (Table 2). Thereby, average values were calculated out of ten AB-model simulations per modelled microenvironment with the data set of S-CA specific weighting factors. Slight differences in model predictions leading to standard deviations are likely caused by AB solver stochasticity and do not have any impact on overall interpretations (see *Results and The Proinflammatory Environment* sections). Therefore, the percentage of inflamed cells for the comparative simulations using invariant weighting factors of 0.01 is based on one representative model simulation.

The weighting factors of the inflammation submodel, i.e., the sensitivity of IL1β and TNF-α mRNA expressions to nutrients, were obtained by using the scaling factor determined by the PN-system ( $\vartheta_{1}=28.7)$ . Note that $\theta_{S}^{CA}$ might become larger than 1, since $\omega_{IL1\beta }$ and $\omega_{TNF-\alpha }$ are not part of the PN-system. Required x-fold mRNA expressions to obtain the weighting factors were received out of both the literature and the *in vitro* experiments of the current study (see *Results and Experimental Results and System of Interest* sections) (Table 3).

**TABLE 3:** Individual weighting factors for the inflammation submodel. ϵ : x-fold mRNA expression, $\theta_{S}^{CA}:$ cell activity and stimulus-specific weighting factor.

Stimulus	mRNA	ϵ	f(ϵ)	$\theta_{S}^{\mathrm{CA}}$	Source	Cell type
Glc	IL1β	NS, act	—	0.01	Actual study	bovine
	TNF-α	NS, act	—	0.01	Actual study	bovine
pH	IL1β	81	81.0000	2.8223	Gilbert et al. (2016)	human
	TNF-α	NS, act	—	0.01	Gilbert et al. (2016)	human

NS: not significant. act: activating.

## Results

### Experimental Results and System of Interest

**FIGURE 6:**
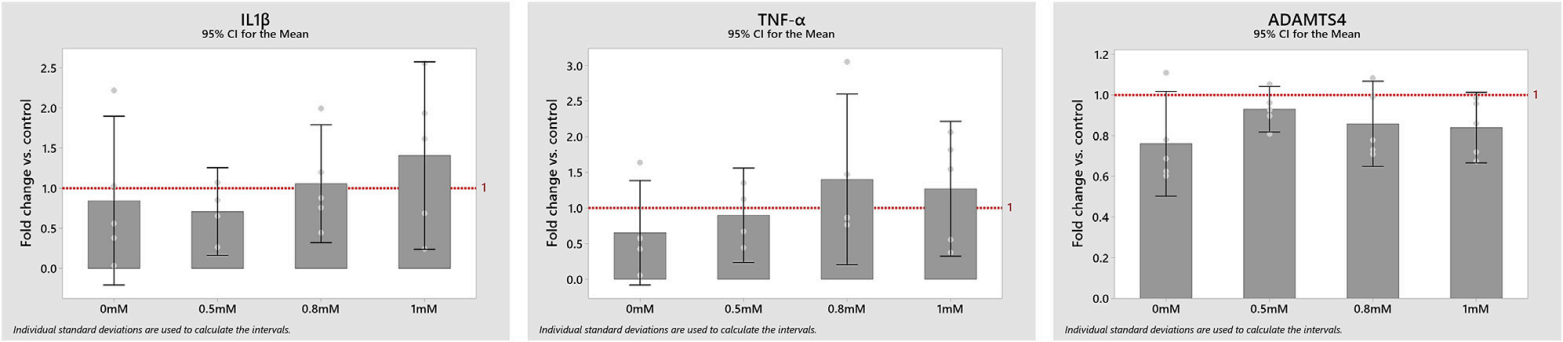
mRNA expression of the proinflammatory cytokines IL1β and TNF-α and the protease ADAMTS4 at 0, 0.5, 0.8 and 1 mM glucose concentrations compared to control (1-fold). Data is displayed as mean values with a corresponding 95% confidence interval and individual values (round dots).

The complete or partial deprivation of glc did not have any statistically significant effect on the mRNA expressions IL1β, TNF-α and ADAMTS4 (Figure 6). Yet, all measured mRNA expressions tended to decrease under complete glc deprivation. Results for IL1β and TNF-α mRNA expressions at 0.5 mM glc were based on four donors instead of five, due to experimental issues.

**FIGURE 7:**
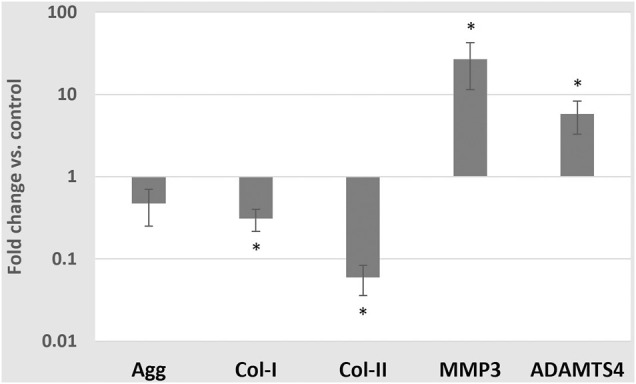
Average mRNA expressions (logarithmic scale) and standard deviations of extracellular matrix proteins and proteases after exposing cells to 10 ng/ml TNF-α, 5 mM glc and pH 7.4. *: significantly (*p* < 0.05) different from control (1-fold).

In contrast, medium enrichment with 10 ng/ml TNF-α caused a significant change in the mRNA expressions of Col-I (0.31 ± 0.09 -fold), Col-II (0.06 ± 0.02 -fold), ADAMTS4 (5.77 ± 2.50 -fold) (*p* < 0.01) and MMP3 (26.85 ± 15.43 -fold) (all *p* < 0.05), but no significant change in the mRNA expression of Agg (0.47 ± 0.22 -fold) (*p* = 0.076) (Figure 7).

**FIGURE 8:**
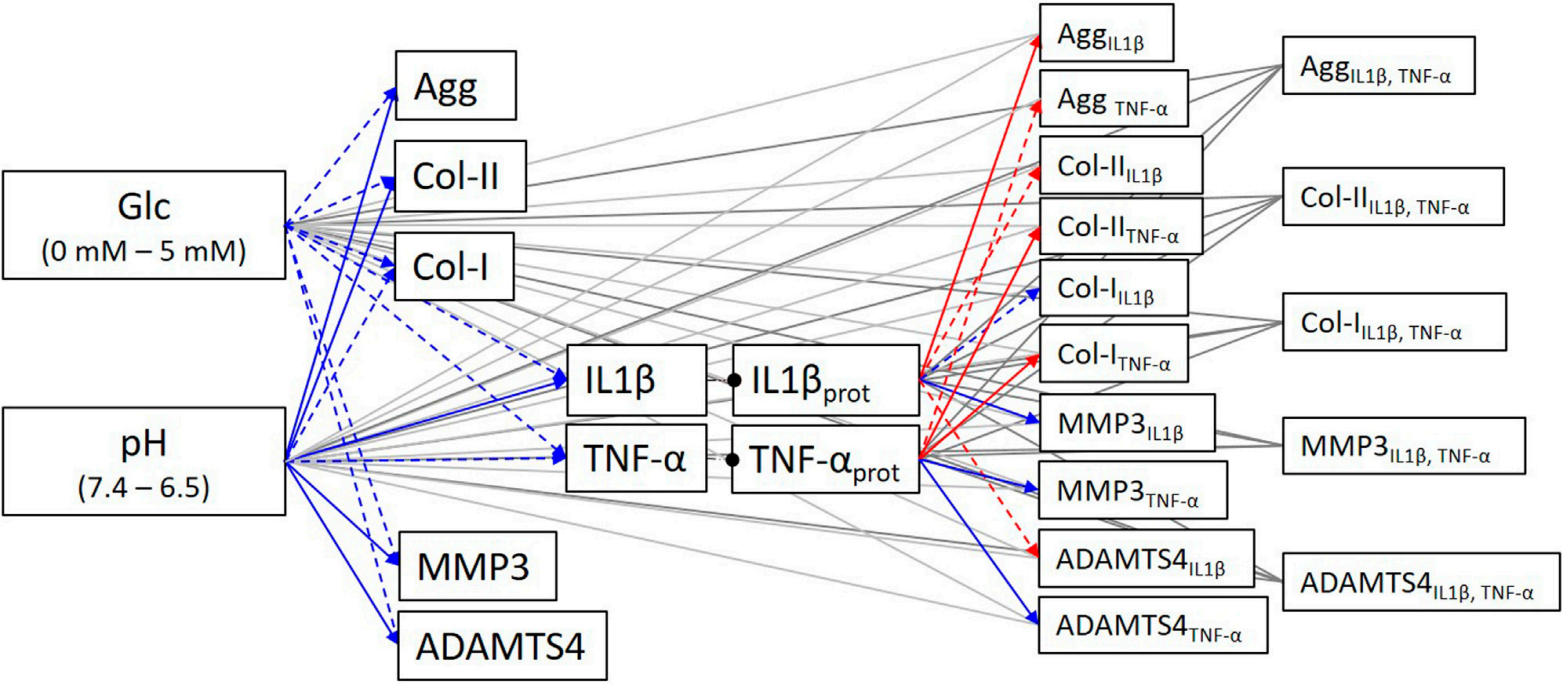
Completed system of interest according to additional experimental data. Blue arrows: activating links; Red arrows: inhibiting links; Dashed arrows: statistically non-significant tendencies.

The obtained experimental measurements led to complete the PN network description of the system of interest, with all activating and inhibiting links (Figure 8).

### *In Silico* Predictions

#### The Proinflammatory Environment

**FIGURE 9:**
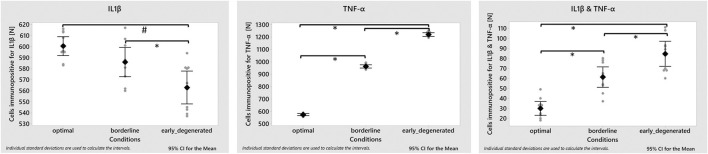
Model predictions for an inflammatory environment within an optimal, borderline and early degenerated nutritional environment. Average amount of inflamed cells **(square)** with corresponding 95% confidence interval and individual values **(grey dots)** (*n* = 10 simulations per condition). ANOVA test showed significant differences between conditions in the three groups studied (*p*-value < 0.001). Post-hoc analysis showed that the number of cells immunopositive for IL1β were significantly lower in the degenerated conditions compared with the optimal and borderline conditions (*p*-value 0.000 and 0.017, respectively). On the other hand, in the case of cells immunopositive for TNF-α and for IL1β&TNF-α, significant differences were observed between the three conditions (*p*-value < 0.001).

The average percentage of cells immunopositive for IL1β (i.e., the sum of cells immunopositive for only IL1β and of both, IL1β&TNF-α) was around 16%, in all three simulated microenvironments, i.e., 15.76 ± 0.11% under optimal; 16.18 ± 0.14% under borderline; 16.23 ± 0.16% under early degenerated conditions. The percentage of TNF-α inflamed cells rose from around 15% under optimal to 26% under borderline up to 33% under early degenerated conditions. Model predictions for cells immunopositive for both, IL1β&TNF-α rose from approximately 1% under physiological to around 2% under early degenerated conditions. The numbers of inflamed cells for TNF-α only, IL1β only or for both TNF-α&IL1β are displayed in Figure 9 for each nutrient condition.

The use of invariant weighting factors of 0.01 led to a cell immunopositivity for IL1β ranging from around 19% for optimal conditions to 35% for borderline and early degenerated conditions. TNF-α immunopositivity did not change, since S-CA specific weighting factors to determine TNF-α have a value of 0.01 (Table 3).

#### Cell Activity

**FIGURE 10:**
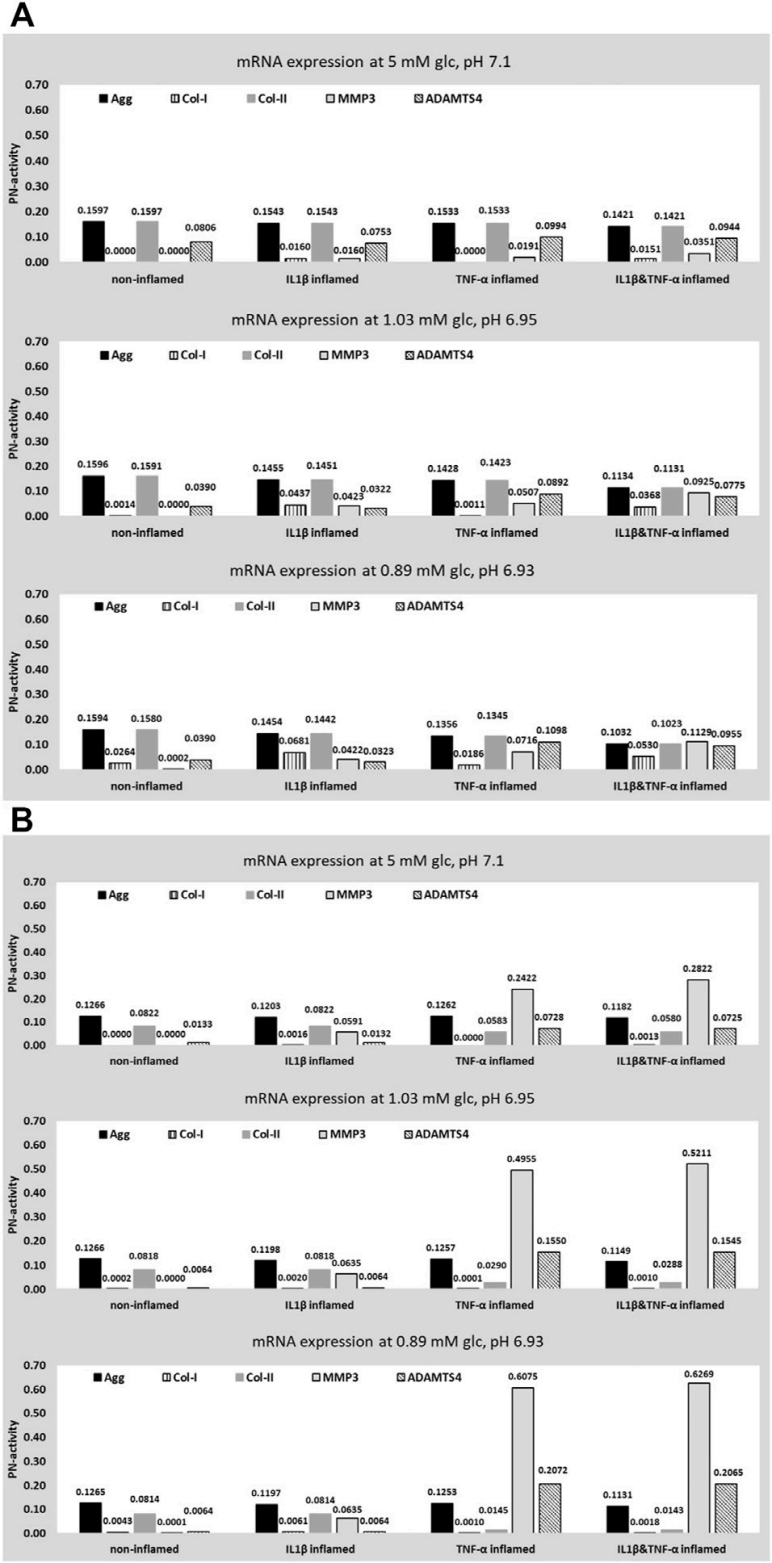
Prediction of five target mRNA expressions for four different proinflammatory cell states. Values were obtained for optimal, borderline and early degenerated, nutritional stimulus combinations. Data was obtained for two sets of weighting factors; an invariant weighting factor of 0.01 **(A)** and an individual weighting factor **(B)**.

Using invariant weighting factors, predicted CA profiles of different inflammatory CS are similar under optimal nutritional conditions, leading to a higher variation under progressively adverse nutrient environments (Figure 10A, from top to bottom). The PN-activity for ADAMTS4 is generally elevated throughout all CA profiles. PN-activities of Agg and Col-II are the same or similar within a CA profile.

In contrast, an application of S-CA specific weighting factors leads to distinct CA profiles for different inflammatory CS and different nutritional conditions (Figure 10B). This includes a pronounced difference between the CA profiles of IL1β and TNF-α inflamed cells, standing out in particular by an elevated protease mRNA expression under the influence of TNF-α (Figure 10B). The predicted PN-activity of ADAMTS4 is lower in non-inflamed and IL1β inflamed cells (Figures 10B vs 10A). Within individual CA profiles, Col-II is generally predicted to be lower than Agg due to an integration of S-CA specific weighting factors (Figures 10B vs 10A). CA profiles of cells immunopositive for both TNF-α&IL1β are similar to the ones of TNF-α inflamed cells (Figure 10B).

## Discussion

### Experimental Results and System of Interest

The need for experimental research was defined by specific *in silico* requirements, which reflects a novelty of this approach. As a consequence, rather unexplored relationships between nutrition-related stimuli and proinflammatory cytokine mRNA expressions were investigated. Measurements suggested non-significant effects of glc variations on the tackled proinflammatory cytokines and on ADAMTS4 (Figure 6). Thus (partial) glc deprivation might not directly trigger enhanced proinflammatory conditions, even though both factors coexist under progressive degeneration (Le Maitre et al., 2005; Le Maitre et al., 2007; Ruiz Wills et al., 2018).

Furthermore, experimental results could not confirm major differences in mRNA expressions at 0.8 mM compared to 1 mM glc concentration. This result suggests that 0.8 mM glc derived from FE predictions (Ruiz Wills et al., 2018) with early degenerated cartilage endplate, might not stand for a relevant nutritional stress for the cells. Arguably, a drop of pH (around 6.9) predicted by the aforementioned FE simulations was not imposed in the experimental setup. The reason for this was that our experiments aimed to provide information about the effect of the variation of a single stimulus at once on a CA, in order to incorporate the measured data in the parallel network model. However, we acknowledge the importance of accessing experimental data with crossed variations of the micro-environmental conditions. Furthermore, general limitations of the experimental part of this study, especially the small sample size, might have masked possible effects. However, for this modelling approach, even non significant variations of mRNA expressions were exploited. The underlying reason was that the chronicity of marginal changes in cell responses might play an important role in intervertebral disc degeneration. Such marginal changes, however, might be masked in experimental research due to pronounced standard deviations and tendentially low sample sizes. Eventually, the impact of experimentally determined significances was regulated by the S-CA specific weighting factors (see *Determination of Weighting Factors* section).

Significant catabolic shifts in CA were observed due to a TNF-α enriched culture medium. This was not surprising, as strong catabolic shifts in cell responses are generally attributed to TNF-α (Purmessur et al., 2013). Catabolic cell responses under the influence of TNF-α could be confirmed for concentrations as low as 1 ng/ml (Séguin et al., 2005). In the current study, a proinflammatory cytokine concentration of 10 ng/ml was applied to facilitate comparability with data from IL1β stimulation (Le Maitre et al., 2005). Although such concentrations might be hyper physiological with physiological levels of TNF-α possibly rather being in the order of pg/ml than ng/ml (Takahashi et al., 1996; Gawri et al., 2014; Zou et al., 2017), they are commonly used *in vitro* to model a pronounced and measurable cell response, even with short stimulation periods. Hence, current predictions about the impact of inflammation on a CA might be disproportionate compared to non-inflamed cell responses. Furthermore, this study used bovine NP cells as a model for non-degenerated human NP cells. This was done before (Rinkler et al., 2010), but, of course, it contains a certain uncertainty regarding the translation of findings between different species. Arguably, because differences between the respective responses of cells of degenerated and non-degenerated intervertebral discs are known (Le Maitre et al., 2005; Le Maitre et al., 2008; Le Maitre et al., 2009), the current experiments primarily targeted non-degenerated intervertebral disc cells (human non-degenerated disc cells are difficult to obtain), to inform the computational model. Furthermore, the experiments were conducted at normoxic conditions, which does not reflect the conditions within an intervertebral disc NP. However, this bias was constantly present throughout the experimental setup, and is therefore considered to have not importantly affected the relative effects of different glc concentrations or TNF-α measured in this study.

More knowledge about the cell response to TNF-α exposure at physiological concentrations might be highly relevant for further model developments. This would allow to ideally estimate the effect of proinflammatory cytokine concentrations as continuous functions, as done for nutrition-related stimuli (Baumgartner et al., 2020, Figure 2). This includes an overall confirmation of the catabolic effect of TNF-α under physiological conditions, especially in the light of experimental research with IL1β that showed an anabolic effect on Agg mRNA expression within 0.001–0.1 ng/ml (Phillips et al., 2015).

Eventually, the experimental data obtained by the current experimental research completed the biological data needed to determine evidence-based S-CA relationships and allowed, therefore, to complete the system of interest (Figure 8).

### *In Silico* Predictions

#### The Proinflammatory Environment

Expected percentages of IL1β inflamed cells for non-degenerated and degenerated intervertebral discs were provided from literature. They range around 17%±7% under non-degenerated and 52%±7% under degenerated conditions (Le Maitre et al., 2007). Thereby, the cohort included patients with severely degenerated tissues. Using S-CA specific weighting factors, the range of IL1β immunopositive cells predicted by the model ranges around 16% for all simulated conditions and hereby lies within the range estimated for non-degenerated conditions. The slight decrease of IL1β immunopositivity in simulated early degeneration (Figure 9) is compensated by an increased number of cells immunnopositive for both, IL1β&TNF-α. Hence the overall amount of IL1β inflamed cells slightly rose under progressively adverse nutritional environments, from 15.76 to 16.23%. Nevertheless, the inflammation within degenerated conditions might be underestimated, given that early degeneration did not lead to a stronger catabolic shift than varying regions within the NP within a non-degenerated NP. In contrast, without considering individualized weighting factors, the values for borderline and early degenerated conditions, i.e. 35% of NP cells immunopositive for IL1β, might be overestimated, and not enough differentiated between the two conditions.

With regard to TNF-α, expected percentages of inflamed cells range around 16 %±7% in non-degenerated and 31%±5% in degenerated conditions (Le Maitre et al., 2007). The model predicted ranges of proinflammatory cytokines of around 15% under optimal, around 26% under borderline and around 33% under early degenerated conditions. Hence, the percentage of TNF-α immunopositive cells under optimal conditions lied within the expected range, whilst the TNF-α immunopositivity under borderline and early degenerated conditions was considered as a clear overestimation. TNF-α is assumed to be an aggressive mediator in catabolic cell responses (Purmessur et al., 2013), and an immunopositivity for TNF-α of 26 and 33%, respectively, of the NP cells close to the mid transversal plane might suggest accelerated local degenerations. With this regard, it must be considered that current predictions of TNF-α rely on pH and glc that both were found to have a non-significant effect (Table 3). Accordingly, S-CA specific weighting factors coincide with invariant weighting factors (i.e. 0.01). Hence, *in silico* predictions of the proinflammatory environment reflect the previous findings that nutrient-environments alone are not sufficient to accurately predict inflammation (*Experimental Results and System of Interest* section). A first step to tackle such limitations is an integration of direct mechanotransduction effects into the model with a subsequent evaluation of the model performance (please check *Cell Activity* section. with this regard).

In contrast to *in silico* methodologies that consider vast network interactions including many (sub) cellular components, this approach only considers relatively few, key relevant external stimuli to estimate overall cell responses at a multicellular level. Hence, instead of using a bottom-up modelling approach to estimate current CA, experimental findings are used to directly link environmental stimulus perturbations to a final CA. Therefore, it is crucial to use external stimuli that are shown to influence the tackled CA. In contrast to tissue proteins or proteases, nutrient-related stimuli alone do not have a determinant impact on proinflammatory cytokine regulations. As a consequence, the model responded with an inaccurate prediction of inflammatory parameters. Hence, this modelling approach seems to be able to sort out the critical characteristics of multifactorial environments to accurately capture a CA. At the same time, it allows high-level and directional modelling, which is important for proper network model interpretations in the light of available evidence.

**FIGURE 11:**
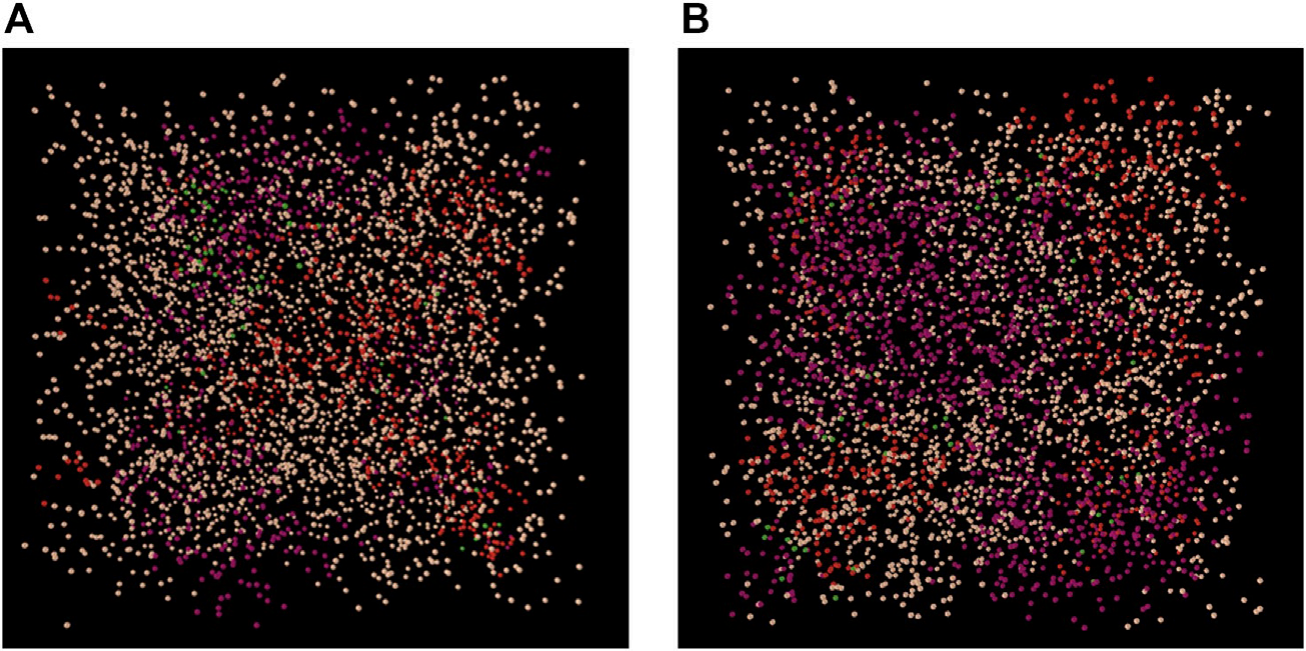
Examples of a inflammatory environments within the 3D Agent-based model under optimal **(A)** and early degenerated **(B)** nutrition conditions. Cell clusters: red; cells immunopositive for IL1β, purple; cells immunopositive for TNF-α; green: cells immunopositive for both, IL1β&TNF-α.

As for the visualization of immunopositivity within the 3D AB-model environment (Figure 11), it was assumed that immunopositive cells were arranged in clusters. Thereby, the location of each cluster was randomly set and the cell number forming each cluster was determined according to the proximity of the cells. Thus, the proinflammatory environment is different for every new model setup. The computational cost to setup the proinflammatory environment ranges around 8 min on an “ordinary” personal computer [in this study: 16 GB RAM, Intel^(R)^ Core^TM^ i7-7500U CPU @ 2.70 GHz (dual core)]. To our knowledge, this is the first approach that provides insights on how a proinflammatory environment might look like within the NP. The idea of immunopositive clusters arise from a combined effect of paracrine stimulation (Phillips et al., 2013; Phillips et al., 2015), short half-life of proinflammatory cytokines and low, diffusion-dependent travel velocities. Due to a lack of data, these simulated clusters of inflammatory environments could not yet be experimentally validated. Arguably, the initial assumption of an independent seeding of IL1β and TNF-α cell clusters might be revised in future model developments, because of the mutual stimulatory effects between TNF-α and IL1β, e.g., the effect of TNF-α on IL1β mRNA expression (Supplementary Material S2). Hence, the number of cells immunopositive for both IL1β&TNF-α might be underestimated. More experimental data about the inflammatory state of NP cells would be needed to better approximate the prediction of proinflammatory intervertebral disc environments. The decreasing costs of transcriptomic and proteomic studies may soon lead to a more comprehensive knowledge about the distribution and type of immunopositive cells within the NP. Moreover, additional information about the response of NP cells to microenvironmental cues represents relevant input data for the herein described model. While these data might help to complement/refine network models (Melas et al., 2014), their interpretation can further benefit from the current modelling approach that uniquely integrates multiple S-CA relationships.

#### Cell Activity

This novel methodological approach allows to tackle regional heterogeneities within the NP, which complements information from experimental research that usually obtains homogenic values for the NP as a whole. Such spatial- and CS-specific CA profiles (Figure 10) are defined by the (local) multifactorial environment and are the result of the interaction of three factors: the sensitivity to a stimulus concentration ( $x_{S}^{CA}$ ), the sensitivity to a stimulus type $(\theta_{S}^{CA}$ ) and their integration through the PN-equation. Hence, PN-activities are constant for constant nutritional boundary conditions and can be obtained within seconds with the “ordinary” personal computer used in this study.

This work focuses on the approximation and the final effect of the stimulus type, which is described with weighting factors. Hence, results are discussed focusing on the impact of weighting factors.

Without the integration of S-CA specific weighting factors, the CA profiles are largely defined by $x_{S}^{CA}$ . This explains why CA profiles look similar in Figure 10A. Hence, the impact of different types of inflammation can only moderately be reflected (Figure 10A, first row) and the effect of CS on CA profiles increases with a progressively adverse nutrient environment (i.e., from the first to the last row, Figure 10A). On the one hand, changes due to nutrient deprivation are small, which coincides with the slow progress of intervertebral disc degeneration, i.e., the chronicity over time might be a major risk factor to eventually compromise the tissue integrity. On the other hand, small variations between different CS would not reflect the strong effects of proinflammatory cytokines on tissue proteins and proteases as suggested by experimental findings (e.g., Figure 7, Le Maitre et al., 2005; Purmessur et al., 2013).

Thanks to an integration of S-CA specific weighting factors, more pronounced differences between CA profiles were predicted and the results show an improved, qualitative agreement with experimental findings which will subsequently be illustrated. Thereby, neither results from CA profiles with TNF-α implication of borderline nutrient environments (situated in Figure 10B, middle row) nor CA profiles with either TNF-α or IL1β implication under early degenerated nutrient-conditions (situated in Figure 10B, last row) will be used for argumentation as a (possible) under- and overestimation of proinflammatory cytokines (see *The Proinflammatory Environment* section) affect corresponding CA-profiles (see Figure 5A).

An implementation of an S-CA specific weighting factor predicts a highly anabolic CA profile of non-inflamed cells. Hence, the ADAMTS4 mRNA expression that was enhanced without the consideration of a weighting factor was decreased (Figures 10A vs 10B, first rows). A low ADAMTS4 mRNA expression coincides with low ADAMTS4 levels in the intervertebral disc NP (Molinos et al., 2015). Compared to the non-inflamed CA-profile, a moderate catabolic shift was predicted for IL1β inflamed cells, reflected by a slow downregulation of Agg and an upregulation of MMP3 and Col-I (Figure 10B). A moderate catabolic shift goes along with the potential role of IL1β in the normal homeostasis of the intervertebral disc (Le Maitre et al., 2007). Likewise, the pronounced catabolic shift due to TNF-α, reflects a previously described rather aggressive impact of TNF-α on CA (Purmessur et al., 2013). Eventually, cells inflamed with both, TNF-α&IL1β generally show a similar, but slightly more catabolic behavior than cells only inflamed with TNF-α. This prediction might be quite conservative, and possibly reflects the need for an incorporation of cross-effects among stimuli. However, few is known so far about cross-effects of different stimuli with regard to mRNA expressions. To our best knowledge, cross-effects were only particularly mentioned with regard to cell viability, where a combination of low pH and a zero glc environment was found to cause more cell death than it would be expected by a simple addition of both individual effects (Bibby and Urban, 2004). The modelling technique presented here can infer, however, on parallel effects. Should nonlinearities of these parallel effects be demonstrated experimentally, new experiment-based functions could be incorporated in the network to eventually reflect cross effects. For example, this could be achieved by formulating the currently constant weighting factors as variables, to let them vary within a predefined range in function of the concentration of other stimuli.

Independently of the CS, an integration of S-CA specific weighting factors led to a generally lower mRNA expression of Col-II, compared to Agg within the same CA profile, whilst for invariant weighting factors same or very similar mRNA expressions of Agg and Col-II were predicted (Figures 10A vs 10B). For example, in case of optimal, nutrient conditions of non-inflamed cells, both Agg and Col-II are maximally activated with a PN-activity of 0.1266 and 0.0822, respectively (Figure 10B, first row). A prediction of a lower, maximal expression of Col-II is in agreement with the tissue composition of the NP, where Agg is more abundant (e.g., reviewed by Baumgartner et al., 2021) and has a faster turnover than Col-II in the (non-degenerated) NP (Sivan et al., 2006; Sivan et al., 2008). This interpretation is valid if it is assumed that 1) the amount of mRNA expression is (largely) proportional to the amount of tissue proteins and 2) that the maximum cell activity of Agg and Col-II mRNA expression is quantitatively similar.

Within this methodological approach, PN-activities are defined with four decimals. Such a numerical precision contrasts with highly varying mRNA expressions among donors and consequently among different studies. While the relevance of this precision has been introduced in the methods, one may question whether it would lead to some over-interpretations of the calculation results. Yet, it is important to highlight that adding numerical uncertainty, e.g., on the high resolution weighting factors, shall not alter qualitatively the predicted CA and the interpretation thereof, under specific simulated environments.

This modelling approach used a determined set of biological data. Experimental findings, however, are sensitive to the experimental setup, including cell types (e.g., human vs. animal), passage numbers, 2D or 3D cultures or time points, at which mRNA expressions were obtained. Given that this network modelling approach is highly evidence-based, discrepancies resulting from experimental differences would consequently be reflected within model results. With this regard, effort was made to use: 1) experimental data that is in overall consensus with widely accepted assumptions of NP cell responses (e.g., a general catabolic effect under rising acidity); 2) studies with human cell culture data rather than animal cells, 3) the measurement of as much required data as possible out of a same experimental study and 4) data from 3D cultures rather than 2D cultures. However, proper integration of possible variations in mRNA expressions at different culture times would become possible if a standardized history of mRNA read-outs is integrated to the experimental protocols for all stimuli. With this regard, focus was set to develop a model design that allows for a straightforward exchange of biological input data as soon as better suitable data is available. In the light of different sets of biological input data in future and a general presence of limitations, it would be suggested to interpret model results stochastically. Hence, by feeding the model with a variation of sets of experimental data, a final probability of the behavior of NP cells under user-defined conditions could be assessed.

Within the current model, mRNA expressions rather than protein synthesis were considered, according to available, experimental data. Unfortunately, a proportional relationship between mRNA expression and protein synthesis is not granted. Hence, the use of biological input data directly based on protein synthesis might be recommendable as soon as such experimental data is available.

The aim of this methodological approach of data integration based on *in vitro* experiments is to estimate cell responses under native conditions. Accordingly, it could also be applied to improve the interpretation of organ culture models such as presented by Ju et al., 2009; Illien-Jünger et al., 2010; Lang et al., 2018. This includes both, cell culture-based knowledge and cues transmitted to the cells through the tissues. Related to the latter, finite element models can be used to define the multiphysics boundary conditions that tissues would impose on the presented AB and network models, as done to define the nutrient environments for “borderline” and “early degenerated” conditions. The metabolic microenvironments defined for the simulated cell collection took into account the heterogeneous deformation and degeneration status of the intervertebral disc tissues, by simulating daily physical activity (Ruiz Wills et al., 2018). Hence, whereas indirect mechanotransduction phenomena are implicitly considered in the model, direct mechanotransduction phenomena are not. Yet, as the present study demonstrates, new experimental data can be aggregated to approach increasingly reasonable predictions of cell activity. In the same way the model was informed through new experiments about nutritional and pro-inflammatory cell stimulation, the parallel networks can be extended to integrate evidence about direct cell mechano-stimulation effects, which are deemed to be cornerstone (Chan et al., 2011; Neidlinger-Wilke et al., 2012; Fearing et al., 2018; Hodson et al., 2018; Saggese et al., 2018). In this work, a proof of concept was presented that parallel networks were able to secure a reasonable description of the apparent CA due to multifactorial biochemical environments. The present study serves as a basis to tackle the complex problem of direct mechanotransduction in the future.

**FIGURE 12:**
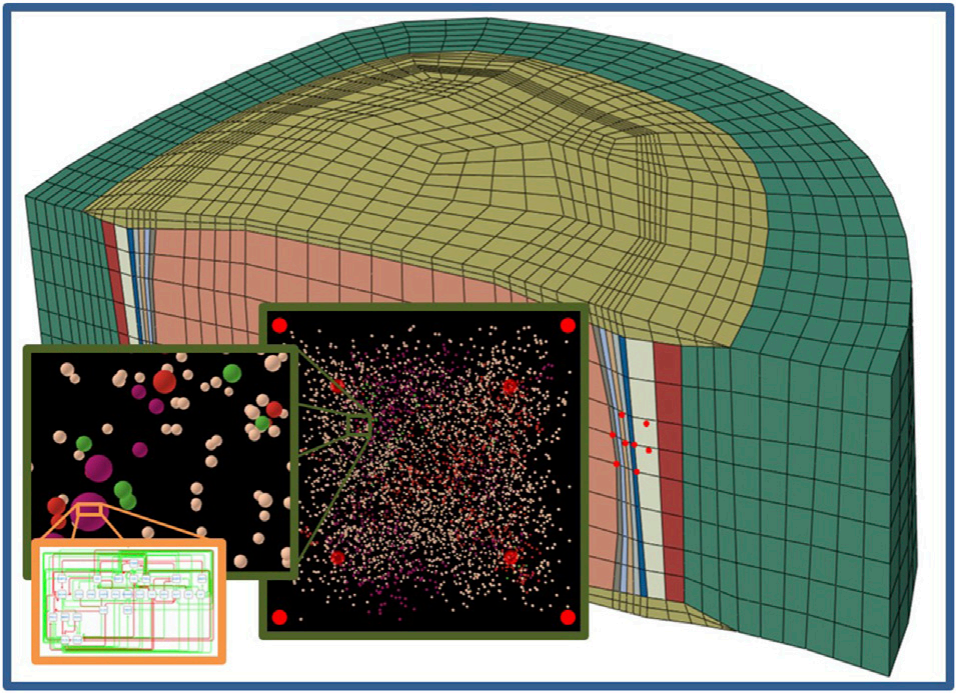
Schematic integration of this modelling approach of the multicellular level into multiscale approaches. Organ/tissue level **(blue frame)**, multicellular level **(green frame)** and subcellular level **(orange frame)**.

This network modelling approach allows to assess local CA based on given environmental conditions at sub-millimetric levels. As mentioned before, our AB input parameters, i.e., local nutrient concentrations, were obtained through the results at the element level of mechanotransport FE simulations (Ruiz Wills et al., 2018). Our predicted CA targets the differential regulation of extracellular matrix turnover that can be used to update the properties of composition-based disc tissue models (Barthelemy et al., 2016; Ruiz Wills et al., 2016), leading to incremental perturbation of local CA in a next iteration of FE-AB simulations. Hence, the present model is deemed to importantly contribute to the development of multiscale modelling approaches to explore intervertebral disc degeneration, where biologically-driven tissue injury includes dynamics over multiple spatial scales (Vergroesen et al., 2015). Likewise, our modelling approach may address the apparent limited capacity of phenomenological mechanobiology models to capture the turnover of intervertebral disc tissues along degeneration (Van Rijsbergen et al., 2018). Furthermore, the networks that control our AB model might be coupled with model developments at lower spatial scales, to integrate mechanistic molecular contributions to intervertebral disc tissue regulation (Figure 12), e.g., in terms of cell regulation pathway signalling, as proposed in osteoarthritis (Melas et al., 2014; Mukherjee et al., 2020).

## Conclusion

This work reflects a multidisciplinary methodology consisting of the integration of experimental (*in vitro*), mathematical (weighting factors, network) and computational (AB) methods, to present an evidence-based enabling technology to approximate the complex multifactorial multicellular environments of the NP. Thereby, biochemical stimuli were considered, and focus was set on estimating proinflammatory environments and cell responses. To duly feed the model, current experimental evidence was completed through new *in vitro* experiments, the results of which were directly incorporated into a novel method to estimate individual CA under multifactorial environments. Remarkably, the results of such integration indicated that differential weighting of the effect of the stimulus concentration was cornerstone to improve the confidence in the simulations.

Experimental results suggest that low glc may not be a main trigger for a catabolic shift in CA. TNF-α, in turn, caused significant catabolic alterations in all mRNA expressions but Agg. The *in silico* model predicted a maximal CA generally lower for Col-II compared to Agg, according to known structural protein turnovers. Low levels of protease mRNA expression were predicted under optimal conditions and non-inflamed and IL1β inflamed cells. Interestingly, the co-existence TNF-α dramatically increases the catabolic shift of CA, with a strong overexpression of key proteases specialised in ECM degradation. Though our number of inflamed cells seemed over-predicted, model simulations indicate that further knowledge and model developments are necessary to capture additional regulators of inflammation. In particular, the incorporation of direct mechanotransduction might be key relevant.

Regarding the prediction of inflammation, the 3D AB model displayed the calculated number of inflamed cell clusters according to the proximity of cells. On the one hand, the assumption that inflammation within the NP is arranged in local cell clusters is based on experimentally known paracrine effects of proinflammatory cytokines, in combination with short half-lives and low diffusivity. On the other hand, such a modelling is a clear asset to quantitatively evaluate the capacity to predict inflammation, for direct comparisons with local biochemical measurements in intervertebral disc specimens. Such quantitative comparisons are instrumental to target specific needs both for model refinements in terms of additional stimuli, and for guided acquisition of new experimental data.

All in all, the current methodology, from network hypothesis to experiments and AB predictions, stands for a unique framework to integrate refined models and new experiments, and to generate, therefore, new contrastable knowledge. Remarkably such process can be fully integrated into multiscale modelling through couplings with FE simulations, to combine both top-down and bottom-up descriptions of the dynamics involved in intervertebral disc degeneration.

At the current stage of development, this model is able to integrate key nutritional and pro-inflammatory cues in 3D multifactorial environments, e.g., enabling more detailed explorations of indirect mechanotransduction phenomena in intervertebral disc degeneration. Further developments will be facilitated by straightforward integration of new biological datasets. Moreover, since the PN mathematical framework is designed to be fully scalable, it allows to integrate any new S-CA relationship based on further experimental evidence.

## Data Availability

The original contributions presented in the study are included in the article/Supplementary Material, further inquiries can be directed to the corresponding author.

## Abbreviations

AB, agent-based; ADAMTS4, A disintegrin and metalloproteinase with thrombospondin motifs 4; Agg, aggrecan; CA, cell activity; Col-I, collagen type I; Col-II, collagen type II; CS, cell status; FE, finite element; glc, glucose; MMP3, matrix metalloproteinase-3; NP, nucleus pulposus; S-CA, stimulus-cell activity; PN, parallel networks.

## References

[B1] Barthelemy V. M. P. van Rijsbergen M. M. Wilson W. Huyghe J. M. van Rietbergen B. Ito K. (2016). A Computational Spinal Motion Segment Model Incorporating a Matrix Composition-Based Model of the Intervertebral Disc. J. Mech. Behav. Biomed. Mater. 54, 194–204. 10.1016/j.jmbbm.2015.09.028 26469631

[B2] Baumgartner L. Reagh J. J. González Ballester M. A. Noailly J. (2020). Simulating Intervertebral Disc Cell Behaviour within 3D Multifactorial Environments. Bioinformatics 37, 1246–1253. 10.1093/bioinformatics/btaa939 PMC8599729 33135078

[B3] Baumgartner L. Wuertz-Kozak K. Le Maitre C. L. Wignall F. Richardson S. M. Hoyland J. (2021). Multiscale Regulation of the Intervertebral Disc: Achievements in Experimental, In Silico, and Regenerative Research. Ijms 22, 703. 10.3390/ijms22020703 PMC7828304 33445782

[B4] Bibby S. R. S. Urban J. P. G. (2004). Effect of Nutrient Deprivation on the Viability of Intervertebral Disc Cells. Eur. Spine J. 13, 695–701. 10.1007/s00586-003-0616-x 15048560 PMC3454063

[B5] Cambria E. Arlt M. J. E. Wandel S. Krupkova O. Hitzl W. Passini F. S. (2020). TRPV4 Inhibition and CRISPR-Cas9 Knockout Reduce Inflammation Induced by Hyperphysiological Stretching in Human Annulus Fibrosus Cells. Cells 9, 1736. 10.3390/cells9071736 PMC7407144 32708074

[B6] Chan S. C. W. Ferguson S. J. Gantenbein-Ritter B. (2011). The Effects of Dynamic Loading on the Intervertebral Disc. Eur. Spine J. 20, 1796–1812. 10.1007/s00586-011-1827-1 21541667 PMC3207351

[B7] Fearing B. V. Hernandez P. A. Setton L. A. Chahine N. O. (2018). Mechanotransduction and Cell Biomechanics of the Intervertebral Disc. JOR Spine 1, 1. 10.1002/jsp2.1026 PMC6296470 30569032

[B8] Gawri R. Rosenzweig D. H. Krock E. Ouellet J. A. Stone L. S. Quinn T. M. (2014). High Mechanical Strain of Primary Intervertebral Disc Cells Promotes Secretion of Inflammatory Factors Associated with Disc Degeneration and Pain. Arthritis Res. Ther. 16, R21. 10.1186/ar4449 24457003 PMC3979109

[B9] Gilbert H. T. J. Hodson N. Baird P. Richardson S. M. Hoyland J. A. (2016). Acidic pH Promotes Intervertebral Disc Degeneration: Acid-Sensing Ion Channel -3 as a Potential Therapeutic Target. Sci. Rep. 6, 1–12. 10.1038/srep37360 27853274 PMC5112591

[B10] Hodson N. W. Patel S. Richardson S. M. Hoyland J. A. Gilbert H. T. J. (2018). Degenerate Intervertebral Disc‐like pH Induces a Catabolic Mechanoresponse in Human Nucleus Pulposus Cells. JOR Spine 1, e1004. 10.1002/jsp2.1004 31463436 PMC6711490

[B11] Hoy D. March L. Brooks P. Blyth F. Woolf A. Bain C. (2014). The Global burden of Low Back Pain: Estimates from the Global Burden of Disease 2010 Study. Ann. Rheum. Dis. 73, 968–974. 10.1136/annrheumdis-2013-204428 24665116

[B12] Illien-Jünger S. Gantenbein-Ritter B. Grad S. Lezuo P. Ferguson S. J. Alini M. (2010). The Combined Effects of Limited Nutrition and High-Frequency Loading on Intervertebral Discs with Endplates. Spine 35, 1744–1752. 10.1097/BRS.0b013e3181c48019 20395884

[B13] Johnson Z. Schoepflin Z. R. Schoepflin Z. Choi H. Shapiro I. Risbud M. (2015). Disc in Flames: Roles of TNF-α and IL-1β in Intervertebral Disc Degeneration. eCM 30, 104–117. 10.22203/eCM.v030a08 26388614 PMC4751407

[B14] Jünger S. Gantenbein-ritter B. Lezuo P. Alini M. Ferguson S. J. Ito K. (2009). Effect of Limited Nutrition on *In Situ* Intervertebral Disc Cells under Simulated-Physiological Loading. Spine 34, 1264–1271. 10.1097/brs.0b013e3181a0193d 19455001

[B15] Krupkova O. Sekiguchi M. Sekiguchi M. Klasen J. Hausmann O. Konno S. (2014). Epigallocatechin 3-gallate Suppresses Interleukin-1β-Induced Inflammatory Responses in Intervertebral Disc Cells *In Vitro* and Reduces Radiculopathic Pain in Rats. eCM 28, 372–386. 10.22203/eCM.v028a26 25422948

[B16] Lang G. Liu Y. Geries J. Zhou Z. Kubosch D. Südkamp N. (2018). An Intervertebral Disc Whole Organ Culture System to Investigate Proinflammatory and Degenerative Disc Disease Condition. J. Tissue Eng. Regen. Med. 12, e2051–e2061. 10.1002/term.2636 29320615

[B17] Le Maitre C. Freemont A. J. Hoyland J. (2005). The Role of Interleukin-1 in the Pathogenesis of Human Intervertebral Disc Degeneration. Arthritis Res. Ther. 7, R732–R745. 10.1186/ar1732 15987475 PMC1175026

[B18] Le Maitre C. Hoyland J. Freemont A. J. (2007). Catabolic Cytokine Expression in Degenerate and Herniated Human Intervertebral Discs: IL-1β and TNFα Expression Profile. Arthritis Res. Ther. 9, R77. 10.1186/ar2275 17688691 PMC2206382

[B19] Le Maitre C. L. Frain J. Fotheringham A. P. Freemont A. J. Hoyland J. A. (2008). Human Cells Derived from Degenerate Intervertebral Discs Respond Differently to Those Derived from Non-degenerate Intervertebral Discs Following Application of Dynamic Hydrostatic Pressure. Biorheology 45, 563–575. 10.3233/BIR-2008-0498 19065005

[B20] Le Maitre C. L. Frain J. Millward-Sadler J. Fotheringham A. P. Freemont A. J. Hoyland J. A. (2009). Altered Integrin Mechanotransduction in Human Nucleus Pulposus Cells Derived from Degenerated Discs. Arthritis Rheum. 60, 460–469. 10.1002/art.24248 19180480

[B21] Likhitpanichkul M. Torre O. M. Gruen J. Walter B. A. Hecht A. C. Iatridis J. C. (2016). Do mechanical Strain and TNF-α Interact to Amplify Pro-inflammatory Cytokine Production in Human Annulus Fibrosus Cells? J. Biomech. 49, 1214–1220. 10.1016/j.jbiomech.2016.02.029 26924657 PMC4913356

[B22] Malandrino A. Jackson A. R. Huyghe J. M. Noailly J. (2015). Poroelastic Modeling of the Intervertebral Disc: A Path toward Integrated Studies of Tissue Biophysics and Organ Degeneration. MRS Bull. 40, 324–332. 10.1557/mrs.2015.68

[B23] Maroudas A. Stockwell R. A. Nachemson A. Urban J. (1975). Factors Involved in the Nutrition of the Human Lumbar Intervertebral Disc: Cellularity and Diffusion of Glucose *In Vitro* . J. Anat. 120, 113–130. 1184452 PMC1231728

[B24] Melas I. N. Chairakaki A. D. Chatzopoulou E. I. Messinis D. E. Katopodi T. Pliaka V. (2014). Modeling of Signaling Pathways in Chondrocytes Based on Phosphoproteomic and Cytokine Release Data. Osteoarthritis and Cartilage 22, 509–518. 10.1016/j.joca.2014.01.001 24457104

[B25] Mendoza L. Xenarios I. (2006). A Method for the Generation of Standardized Qualitative Dynamical Systems of Regulatory Networks. Theor. Biol. Med. Model. 3, 13. 10.1186/1742-4682-3-13 16542429 PMC1440308

[B26] Millward-Sadler S. J. Costello P. W. Freemont A. J. Hoyland J. A. (2009). Regulation of Catabolic Gene Expression in normal and Degenerate Human Intervertebral Disc Cells: Implications for the Pathogenesis of Intervertebral Disc Degeneration. Arthritis Res. Ther. 11, R65. 10.1186/ar2693 19435506 PMC2714110

[B27] Molinos M. Almeida C. R. Caldeira J. Cunha C. Gonçalves R. M. Barbosa M. A. (2015). Inflammation in Intervertebral Disc Degeneration and Regeneration. J. R. Soc. Interf. 12, 20141191. 10.1098/rsif.2014.1191 PMC4528607 26040602

[B28] Mukherjee S. Nazemi M. Jonkers I. Geris L. (2020). Use of Computational Modeling to Study Joint Degeneration: A Review. Front. Bioeng. Biotechnol. 8, 1–12. 10.3389/fbioe.2020.00093 32185167 PMC7058554

[B29] Nachemson A. (1969). Intradiscal Measurements of Ph in Patients with Lumbar Rhizopathies. Acta Orthopaedica Scand. 40, 23–42. 10.3109/17453676908989482 4312806

[B30] Neidlinger-Wilke C. Mietsch A. Rinkler C. Wilke H.-J. Ignatius A. Urban J. (2012). Interactions of Environmental Conditions and Mechanical Loads Have Influence on Matrix Turnover by Nucleus Pulposus Cells. J. Orthop. Res. 30, 112–121. 10.1002/jor.21481 21674606

[B31] Oliver J. C. Bland L. A. Oettinger C. W. Arduino M. J. McAllister S. K. Aguero S. M. (1993). Cytokine Kinetics in an *In Vitro* Whole Blood Model Following an Endotoxin challenge. Lymphokine Cytokine Res. 12, 115–120. 8324076

[B32] Phillips K. L. E. Chiverton N. Michael A. L. Cole A. A. Breakwell L. M. Haddock G. (2013). The Cytokine and Chemokine Expression Profile of Nucleus Pulposus Cells: Implications for Degeneration and Regeneration of the Intervertebral Disc. Arthritis Res. Ther. 15, R213. 10.1186/ar4408 24325988 PMC3979161

[B33] Phillips K. L. E. Cullen K. Chiverton N. Michael A. L. R. Cole A. A. Breakwell L. M. (2015). Potential Roles of Cytokines and Chemokines in Human Intervertebral Disc Degeneration: Interleukin-1 Is a Master Regulator of Catabolic Processes. Osteoarthritis and Cartilage 23, 1165–1177. 10.1016/j.joca.2015.02.017 25748081

[B34] Purmessur D. Walter B. A. Roughley P. J. Laudier D. M. Hecht A. C. Iatridis J. (2013). A Role for TNFα in Intervertebral Disc Degeneration: A Non-recoverable Catabolic Shift. Biochem. Biophysical Res. Commun. 433, 151–156. 10.1016/j.bbrc.2013.02.034 PMC3640343 23438440

[B35] Rijsbergen M. v. Van Rietbergen B. Barthelemy V. Eltes P. Lazáry Á. Lacroix D. (2018). Comparison of Patient-specific Computational Models vs. Clinical Follow-Up, for Adjacent Segment Disc Degeneration and Bone Remodelling after Spinal Fusion. PLoS One 13, e0200899–24. 10.1371/journal.pone.0200899 30161138 PMC6116979

[B36] Rinkler C. Heuer F. Pedro M. T. Mauer U. M. Ignatius A. Neidlinger-Wilke C. (2010). Influence of Low Glucose Supply on the Regulation of Gene Expression by Nucleus Pulposus Cells and Their Responsiveness to Mechanical Loading. Spi 13, 535–542. 10.3171/2010.4.SPINE09713 20887152

[B37] Risbud M. V. Shapiro I. M. (2014). Role of Cytokines in Intervertebral Disc Degeneration: Pain and Disc Content. Nat. Rev. Rheumatol. 10, 44–56. 10.1038/nrrheum.2013.160 24166242 PMC4151534

[B38] Ruiz Wills C. Foata B. González Ballester M. Á. Karppinen J. Noailly J. (2018). Theoretical Explorations Generate New Hypotheses about the Role of the Cartilage Endplate in Early Intervertebral Disk Degeneration. Front. Physiol. 9, 1–12. 10.3389/fphys.2018.01210 30283342 PMC6156535

[B39] Sadowska A. Altinay B. Hitzl W. Ferguson S. J. Wuertz-Kozak K. (2020). Hypo-Osmotic Loading Induces Expression of IL-6 in Nucleus Pulposus Cells of the Intervertebral Disc Independent of TRPV4 and TRPM7. Front. Pharmacol. 11, 1–16. 10.3389/fphar.2020.00952 32714187 PMC7341822

[B40] Saggese T. Thambyah A. Wade K. McGlashan S. R. (2018). Differential Response of Bovine Mature Nucleus Pulposus and Notochordal Cells to Hydrostatic Pressure and Glucose Restriction. Cartilage 11, 221–233. 10.1177/1947603518775795 29808709 PMC7097982

[B41] Séguin C. A. Pilliar R. M. Roughley P. J. Kandel R. A. (2005). Tumor Necrosis Factorα Modulates Matrix Production and Catabolism in Nucleus Pulposus Tissue. Spine 30, 1940–1948. 10.1097/01.brs.0000176188.40263.f9 16135983

[B42] Sivan S.-S. Wachtel E. Tsitron E. Sakkee N. Van Der Ham F. DeGroot J. (2008). Collagen Turnover in normal and Degenerate Human Intervertebral Discs as Determined by the Racemization of Aspartic Acid. J. Biol. Chem. 283, 8796–8801. 10.1074/jbc.M709885200 18250164

[B43] Sivan S. S. Tsitron E. Wachtel E. Roughley P. J. Sakkee N. Van Der Ham F. (2006). Aggrecan Turnover in Human Intervertebral Disc as Determined by the Racemization of Aspartic Acid. J. Biol. Chem. 281, 13009–13014. 10.1074/jbc.M600296200 16537531

[B44] Takahashi H. Suguro T. Okazima Y. Motegi M. Okada Y. Kakiuchi T. (1996). Inflammatory Cytokines in the Herniated Disc of the Lumbar Spine. Spine 21, 218–224. 10.1097/00007632-199601150-00011 8720407

[B45] Urban J. P. G. Smith S. Fairbank J. C. T. (2004). Nutrition of the Intervertebral Disc. Spine 29, 2700–2709. 10.1097/01.brs.0000146499.97948.52 15564919

[B46] Veres S. P. Robertson P. A. Broom N. D. (2008). ISSLS Prize Winner: Microstructure and Mechanical Disruption of the Lumbar Disc Annulus. Spine 33, 2711–2720. 10.1097/BRS.0b013e31817bb906 19002077

[B47] Vergroesen P.-P. A. Kingma I. Emanuel K. S. Hoogendoorn R. J. W. Welting T. J. van Royen B. J. (2015). Mechanics and Biology in Intervertebral Disc Degeneration: A Vicious circle. Osteoarthritis and Cartilage 23, 1057–1070. 10.1016/j.joca.2015.03.028 25827971

[B48] Walter B. A. Purmessur D. Likhitpanichkul M. Weinberg A. Cho S. K. Qureshi S. A. (20151976). Inflammatory Kinetics and Efficacy of Anti-inflammatory Treatments on Human Nucleus Pulposus Cells. Spine 40, 955–963. 10.1097/BRS.0000000000000932 PMC4567470 25893355

[B49] Wilensky U. (1999). NetLogo. Available at: http://ccl.northwestern.edu/netlogo/.

[B50] Wills C. R. Malandrino A. Van Rijsbergen M. Lacroix D. Ito K. Noailly J. (2016). Simulating the Sensitivity of Cell Nutritive Environment to Composition Changes within the Intervertebral Disc. J. Mech. Phys. Sol. 90, 108–123. 10.1016/j.jmps.2016.02.003

[B51] Yang X. Wang L. Yuan Z. Zhou P. Chu G. Li B. (2017). Interleukin-1β Induces Metabolic and Reactive Oxygen Species Changes and Apoptosis in Annulus Fibrosus Cells. Int. J. Clin. Exp. Med. 10, 16144–16153.

[B52] Zou J. Chen Y. Qian J. Yang H. (2017). Effect of a Low-Frequency Pulsed Electromagnetic Field on Expression and Secretion of IL-1β and TNF-α in Nucleus Pulposus Cells. J. Int. Med. Res. 45, 462–470. 10.1177/0300060516683077 28173722 PMC5536647

