# Supplementary material for: Evidence-Based Network Modelling to Simulate Nucleus Pulposus Multicellular Activity in Different Nutritional and Pro-Inflammatory Environments
Source: Front Bioeng Biotechnol. 2021 Nov 10;9:734258. doi: 10.3389/fbioe.2021.734258 (PMC8631496; doi:10.3389/fbioe.2021.734258)
Supplement: Supplementary file 1 [file DataSheet1.pdf]

Supplementary Material

1 Cell viability

A tendency of lower cell viability is observed as nutritional stress increases, although this change is not significant, whereas an enhanced TNF-α level within the culture serum did not reveal any cytotoxic effect (Supplementary Figure 1)

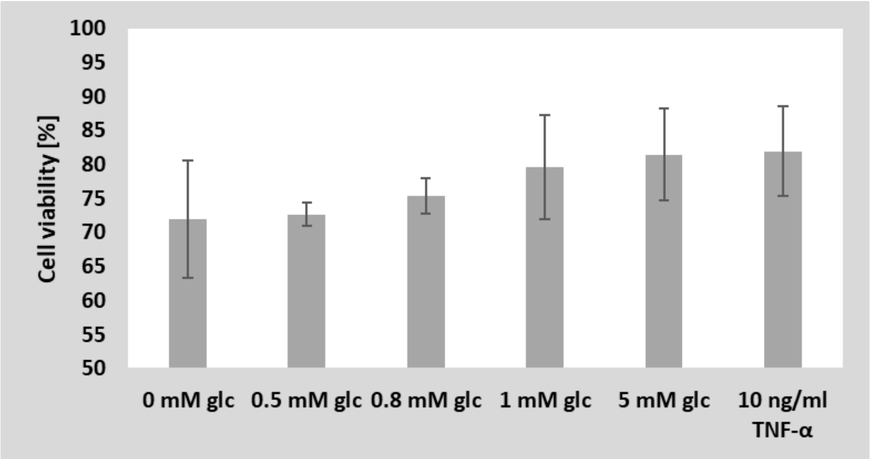

Supplementary Figure 1: Cell viability at different glucose (glc) levels and at exposure to 10 ng/ml TNF-α . Therefore, one alginate bead of each condition was exposed to a 10 µm Calcein AM (CaAM)/1 µM Ethidium Homodimer (EthHD) solution and gently squeezed for analysis under a fluorescence microscope. Each bead was analysed within up to four different regions of the bead, and cells were counted within a predefined area. Evaluation was based on all five donors for each culture condition.

Our findings were consistent with findings in literature. Rinkler et al., 2010 stated a decrease in cell viability on (partial) glucose deprivation, whereas enhanced TNF-α levels within the culture serum did not affect NP cell viability (Li et al., 2017).

2 mRNA expressions of Nucleus Pulposus cells under reduced glucose conditions or TNF-α supply

Significant gene expression modifications are observed under nutritional stress (p=0,039). However, univariate analysis does not show any single mRNA level expression responsible for this difference suggesting a synergistic effect of all of them (Supplementary Figure 2, A). In contrast, the effect of TNF-α (10 ng/ml) on both, TNF-α and IL1β mRNA expression was found to be significant ($p < 0.05$) (Supplementary Figure 2, B).

A

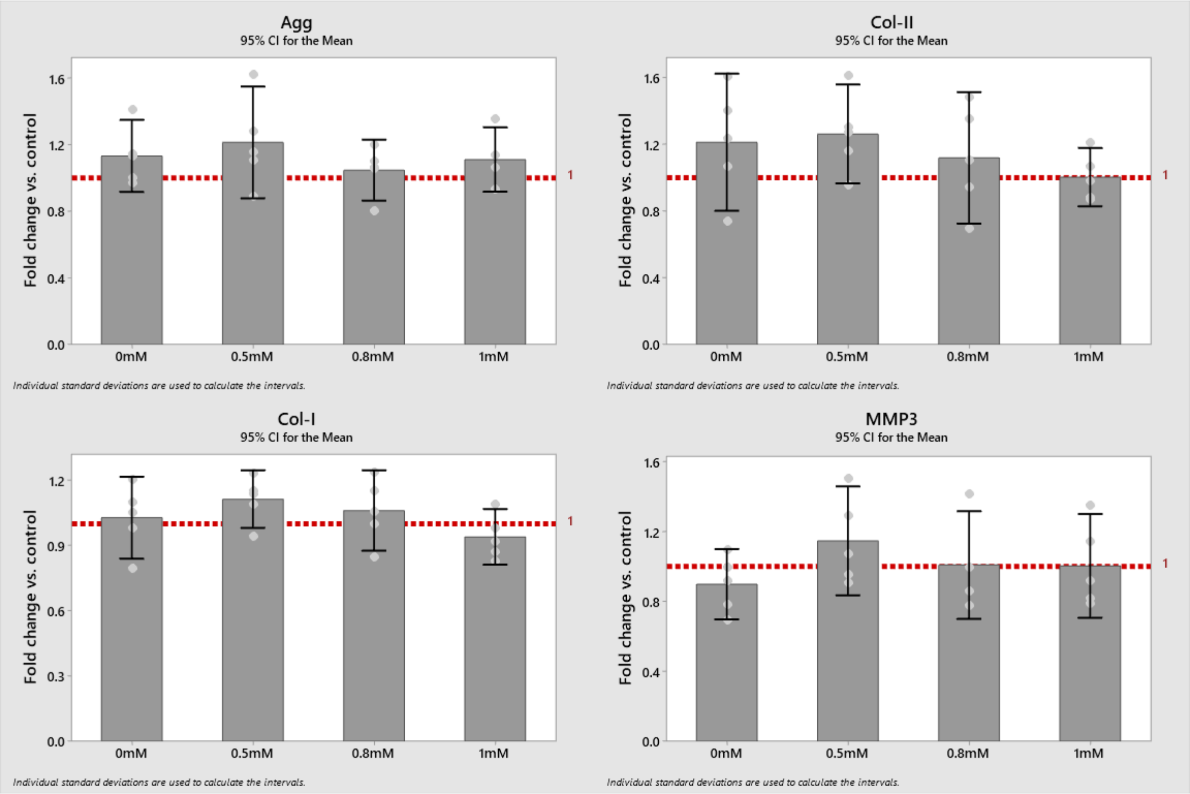

B

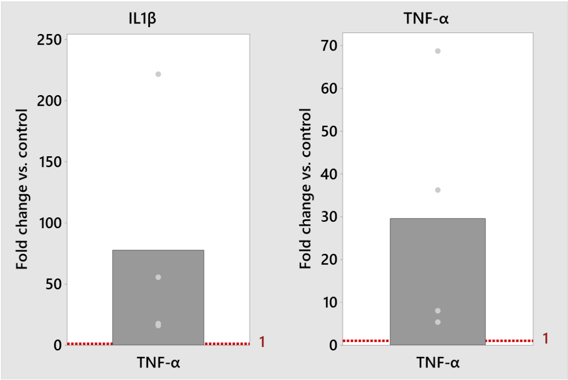

Supplementary Figure 2: A: mRNA expressions of Aggrecan (Agg), Collagen types I and II (Col-I, Col-II) and MMP3 at glucose (glc) concentrations of 0 mM, 0.5 mM, 0.8 mM, 1 mM and 5 mM (control; 1-fold mRNA expression) (biological replicates, n=5). B: IL1β and TNF-α mRNA expressions due to an exposure to 10 ng/ml TNF-α compared to control (5mM glc; 1-fold mRNA expression) (biological replicates, n=4).

Non-significant effects of reduced glucose supply on MMP3 mRNA expressions of bovine NP cells was as well found by Rinkler et al., 2010. However, our study could not confirm significant downregulations of Agg, Col-I and Col-II mRNA expressions of bovine NP cells due to decreases in glc concentrations found by those authors, despite the similar experimental protocol. Previously known stimulatory effects of TNF-α on proinflammatory cytokines (Millward-Sadler et al., 2009; Purmessur et al., 2013) could be confirmed by this study.

3 Sensitivity of a cell to a stimulus concentration

Supplementary Table 1: Functions to relate physiological ranges of stimulus concentrations to a normalized sensitivity of a cell activity to a stimulus concentration. Tackled cell activities: mRNA expressions of proinflammatory cytokines, tissue proteins and proteases.

mRNA expression	Glc	pH
**Agg**	$x_{glc}^{Agg} = \frac{e^{18glc}}{e^{18glc} + 3 * 10^4}$	$6.5 \leq pH \leq 6.892$<br>$x_{pH}^{Agg} = \frac{e^{68(\mathrm{pH}-6.5)}}{e^{68(\mathrm{pH}-6.5)} + 2 * 10^4}$<br>$6.892 < pH \leq 7.4$<br>$x_{pH}^{Agg} = \frac{-e^{32(\mathrm{pH}-6.5)}}{e^{32(\mathrm{pH}-6.5)} + 5.035 * 10^{12}} + 1$
**Col-I**	$0 \leq glc \leq 0.5043$<br>$x_{glc}^{Col-I} = \frac{e^{20glc}}{e^{20glc} + 1.083}$<br>$0.5043 < glc \leq 5$<br>$x_{glc}^{Col-I} = \frac{-e^{28glc}}{e^{28glc} + 3 * 10^{10}} + 1$	$6.5 \leq pH < 7.1207$<br>$x_{pH}^{Col-I} = \frac{-e^{35(\mathrm{pH}-6.5)}}{e^{35(\mathrm{pH}-6.5)} + 6.002 * 10^4} + 1$<br>$7.1207 \leq pH \leq 7.4$<br>$x_{pH}^{Col-I} = \frac{e^{35(\mathrm{pH}-6.5)}}{e^{35(\mathrm{pH}-6.5)} + 1.2376 * 10^{14}}$
**Col-II**	$x_{glc}^{Col-II} = \frac{e^{18*glc}}{e^{18*glc} + 9.4 * 10^4}$	$x_{pH}^{Col-II} = \frac{e^{34(\mathrm{pH}-6.5)}}{e^{34(\mathrm{pH}-6.5)} + 2.845 * 10^4}$
**MMP3**	$x_{glc}^{MMP3} = \frac{-e^{18glc}}{e^{18glc} + 2.283 * 10^4} + 1$	$6.5 \leq pH \leq 6.974$<br>$x_{pH}^{MMP3} = \frac{-e^{48(\mathrm{pH}-6.5)}}{e^{48(\mathrm{pH}-6.5)} + 2.18 * 10^4} + 1$<br>$6.974 < pH \leq 7.4$<br>$x_{pH}^{MMP3} = \frac{e^{20(\mathrm{pH}-6.5)}}{e^{20(\mathrm{pH}-6.5)} + 4.62 * 10^9}$
**ADAMTS4**	$0 \leq glc \leq 1.197$<br>$x_{glc}^{ADAMTS4} = \frac{e^{38glc} * 0.4817}{e^{38glc} + 1 * 10^4}$<br>$1.197 < glc \leq 5$<br>$x_{glc}^{ADAMTS4} = \frac{e^{6glc} * 0.520}{e^{6glc} + 4 * 10^5} + 0.48$	$x_{pH}^{ADAMTS4} = \frac{-e^{44(\mathrm{pH}-6.5)}}{e^{44(\mathrm{pH}-6.5)} + 1 * 10^3} + 1$
**IL1β**	$0 \leq glc \leq 1.4578$<br>$x_{glc}^{IL1\beta} = \frac{e^{55glc}}{e^{55glc} + 1.62507 * 10^{19}}$	$6.5 \leq pH \leq 7.0372$<br>$x_{pH}^{IL1\beta} = \left(\frac{-e^{55(\mathrm{pH}-6.5)}}{(e^{55(\mathrm{pH}-6.5)} + (1.94 * 10^{4})} + 1.036\right) * \frac{1}{1.036}$

	$1.4578 < glc \le 5$<br>$x_{glc}^{IL1\beta} = -\frac{e^{4glc} * 0.648}{e^{4glc} + 1.314 * 10^{6}} + 1.000168$	$7.0372 < pH \le 7.4$<br>$x_{pH}^{IL1\beta} = \left(\frac{-e^{35(\mathrm{pH}-6.5)}}{(e^{35(\mathrm{pH}-6.5)} + (5.4 * 10^{10})} + 1\right) * \frac{1}{28.7}$
**TNF-α**	$0 \le glc \le 0.8134$<br>$x_{glc}^{TNF-\alpha} = \frac{e^{36glc}}{e^{36glc} + 0.135196 * 10^{9}}$<br>$0.8134 < glc \le 5$<br>$x_{glc}^{TNF-\alpha} = \frac{-e^{24glc} * 0.5389}{e^{24glc} + 0.53304 * 10^{11}} + 1.003$	$6.5 \le pH < 6.8042$<br>$x_{pH}^{TNF-\alpha} = \frac{e^{32(\mathrm{pH}-6.5)}}{e^{32(\mathrm{pH}-6.5)} + 0.6474}$<br>$6.8042 \le pH \le 7.1115$<br>$x_{pH}^{TNF-\alpha} = \frac{-e^{68(\mathrm{pH}-6.5)}}{e^{68(\mathrm{pH}-6.5)} + 2.5 * 10^{13}} + 1$<br>$7.1115 \le pH \le 7.4$<br>$x_{pH}^{TNF-\alpha} = \frac{e^{42(\mathrm{pH}-6.5)}}{e^{42(\mathrm{pH}-6.5)} + 6.5 * 10^{15}}$

References

Li, P., Gan, Y., Xu, Y., Song, L., Wang, L., Ouyang, B., et al. (2017). The inflammatory cytokine TNF-α promotes the premature senescence of rat nucleus pulposus cells via the PI3K/Akt signaling pathway. *Sci. Rep.* 7, 1–12. doi:10.1038/srep42938.

Millward-Sadler, S. J., Costello, P. W., Freemont, A. J., and Hoyland, J. A. (2009). Regulation of catabolic gene expression in normal and degenerate human intervertebral disc cells: implications for the pathogenesis of intervertebral disc degeneration. *Arthritis Res. Ther.* 11, R65. doi:10.1186/ar2693.

Purmessur, D., Walter, B. a, Roughley, P. J., Laudier, D. M., Hecht, a C., and Iatridis, J. (2013). A role for TNFα in intervertebral disc degeneration: a non-recoverable catabolic shift. *Biochem. Biophys. Res. Commun.* 433, 151–6. doi:10.1016/j.bbrc.2013.02.034.

Rinkler, C., Heuer, F., Pedro, M. T., Mauer, U. M., Ignatius, A., and Neidlinger-Wilke, C. (2010). Influence of low glucose supply on the regulation of gene expression by nucleus pulposus cells and their responsiveness to mechanical loading. *J. Neurosurg. Spine J Neurosurg Spine* 13, 535–542. doi:10.3171/2010.4.SPINE09713.
